# Observation of optical solitons in PT-symmetric lattices

**DOI:** 10.1038/ncomms8782

**Published:** 2015-07-28

**Authors:** Martin Wimmer, Alois Regensburger, Mohammad-Ali Miri, Christoph Bersch, Demetrios N. Christodoulides, Ulf Peschel

**Affiliations:** 1 Institute of Optics, Information and Photonics, Friedrich-Alexander-Universität Erlangen-Nürnberg, Staudtstraße 7/B2, 91058 Erlangen, Germany; 2 Erlangen Graduate School in Advanced Optical Technologies (SAOT), 91058 Erlangen, Germany; 3 CREOL, College of Optics and Photonics, University of Central Florida, Orlando, Florida 32816–2700, USA; 4 Institute of Solid State Theory and Optics, Friedrich Schiller University Jena, Max-Wien-Platz 1, 07743 Jena

## Abstract

Controlling light transport in nonlinear active environments is a topic of considerable interest in the field of optics. In such complex arrangements, of particular importance is to devise strategies to subdue chaotic behaviour even in the presence of gain/loss and nonlinearity, which often assume adversarial roles. Quite recently, notions of parity-time (PT) symmetry have been suggested in photonic settings as a means to enforce stable energy flow in platforms that simultaneously employ both amplification and attenuation. Here we report the experimental observation of optical solitons in PT-symmetric lattices. Unlike other non-conservative nonlinear arrangements where self-trapped states appear as fixed points in the parameter space of the governing equations, discrete PT solitons form a continuous parametric family of solutions. The possibility of synthesizing PT-symmetric saturable absorbers, where a nonlinear wave finds a lossless path through an otherwise absorptive system is also demonstrated.

## Abstract (web summary)

Parity–time symmetry can impose a stable energy flow in photonic systems with simultaneous amplification and attenuation. Here, Wimmer *et al.* demonstrate optical solitons belonging to a continuous parametric family of solutions in a parity–time-symmetric lattice and observe saturable absorber action.

## Graphical abstract

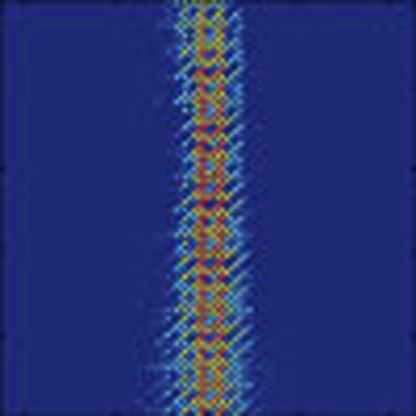

The interplay between energy dissipation and nonlinearity plays a crucial role in many and diverse areas of science ranging from biology and chemistry to thermodynamics^1^. Yet, apart from a few well-studied cases, the interaction of these two processes still remains poorly understood. In optics, for example, such nonlinear gain/loss systems are routinely encountered in laser configurations or in nonlinear active cavities and optical fibres, which are typically described by Ginzburg–Landau-type equations^2^,^3^. Part of the difficulty in analysing such arrangements lies with the fact that the linear eigenvalues of the associated non-Hermitian system are generally positioned in the complex domain. As a result, some of the eigenstates tend to grow uncontrollably and in conjunction with the nonlinearity they force the ensuing dynamics into a chaotic motion. One way to avoid these complications is to ensure that the linear spectrum is completely real, even though the underlying problem itself is non-Hermitian.

An answer to this interesting possibility was given few years ago by Bender and Boettcher when they indicated that a wide class of non-Hermitian Hamiltonians can indeed display altogether real spectra, provided they respect parity-time (PT) symmetry^4^. A necessary condition for establishing this quasi-energy-conserving behaviour is that the associated complex potentials satisfy V*(−*x*)=*V*(*x*). In other words, the real part of the potential must be an even function of position while the imaginary must be antisymmetric^4^,^5^,^6^,^7^. For a given real component of the potential, the eigenvalues of the Hamiltonian remain real, as long as its imaginary counterpart is below a certain critical value, the so-called PT-symmetry-breaking threshold^5^. Above this particular threshold, the spectrum ceases to be entirely real and the respective modes grow instead exponentially.

In photonics, PT-symmetric complex potentials can be readily implemented by symmetrically intermixing gain/loss regions in conjunction with refractive index modulation^8^,^9^,^10^,^11^,^12^. As shown in several studies, PT-symmetric optical arrangements can exhibit several interesting and counterintuitive properties, which are otherwise unattainable in standard configurations^8^,^9^,^10^,^11^,^12^,^13^,^14^,^15^,^16^,^17^,^18^,^19^,^20^,^21^,^22^. These include for example, power unfolding and breaking of the left-right symmetry^8^, abrupt phase transitions^9^,^10^,^11^,^12^, non-Hermitian Bloch oscillations^14^, simultaneous lasing-absorbing^16^ and selective mode lasing in microring resonator systems^23^,^24^. Moreover, unidirectional invisibility^11^,^25^,^26^ and defect states^27^ with unconventional properties have been also demonstrated. Finally, PT-symmetric concepts have also been used in plasmonics and optical metamaterials^28^. Lately, it has been shown that operating close to the exceptional point of a PT-symmetric coupled microring arrangement can significantly affect thermal nonlinearities and Raman lasing^29^. Clearly of interest would be to investigate the role of nonlinearity within the framework of PT-symmetric periodic structures and lattices.

Recently non-reciprocal light propagation and diode behaviour was observed in two coupled PT-symmetric whispering-gallery microcavities with a saturable nonlinearity, thus enabling new possibilities for on chip signal processing^30^,^31^. Yet, the nonlinear response of extended PT-symmetric systems can greatly benefit from the stability offered by a special class of self-localized solutions—the so-called solitons^32^,^33^,^34^. Solitons are thought to be the natural building blocks of any nonlinear system. Apart from preserving their shape during propagation, they exhibit remarkable robustness against external perturbations and they tend to collide with each other in a particle-like manner^33^. While optical solitons have been previously identified in dissipative Ginzburg–Landau settings^35^, their observation in PT-symmetric environments^36^ still remains a challenge in spite of numerous theoretical predictions^36^,^37^,^38^,^39^,^40^,^41^,^42^,^43^,^44^,^45^,^46^,^47^,^48^,^49^. This is partly due to the difficulty of introducing gain/loss and nonlinearity in perfect synergy. Interestingly, this problem can be efficiently circumvented in coupled fibre loop arrangements that happen to be isomorphic to mesh lattices in the time domain^11^. The inherent discreteness of this configuration further adds simplicity^50^,^51^,^52^, and also establishes a link to other non-optical systems like Bose condensates in lattices or electronic excitations along molecular chains.

In this Article, we show that PT-symmetric solitons can be systematically investigated in coupled fibre loop platforms. These non-conservative nonlinear mesh lattices can be utilized to directly study the interplay between nonlinearity and a balanced gain/loss profile. Along these lines, we observe stable soliton entities in lattices with local PT symmetry that fully conform to theoretical predictions. In addition, the existence of similar self-trapped states is also demonstrated in mesh periodic structures with global PT symmetry. In all cases, these solitons are found to belong to a continuous family of solutions^37^,^39^—which is not typically the case for dissipative solitary waves^35^,^53^,^54^,^55^,^56^,^57^. Finally, we show that this class of discrete solitons^34^,^58^,^59^,^60^,^61^ can evade instabilities. In this case, a loss in the system tends to suppress low power signals, while it is overpowered by nonlinearity, thus leading to a saturable absorber action.

## Results

### Experimental setup and theoretical model

**Figure 1:**
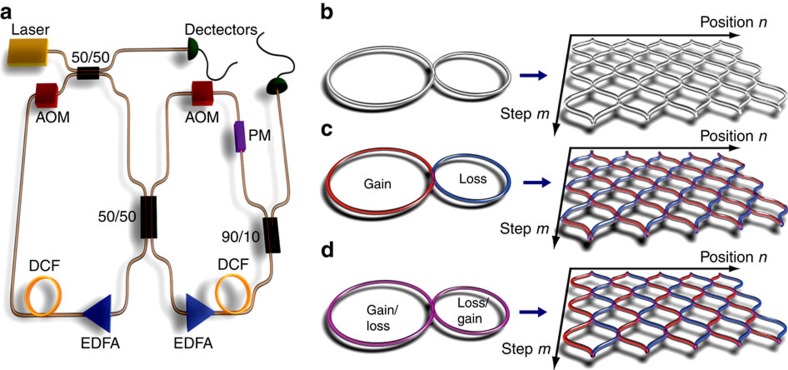
Experimental realization of the discrete mesh lattice. Two coupled fibre loops (**a**) of different length are used to implement the mesh lattices displayed in (**b**–**d**). Four kilometres of dispersion compensating fibres (DCF) are inserted into the loops to amplify the nonlinear phase shift. The phase and amplitude of the signals are controlled by a phase modulator (PM) and acousto-optical modulators (AOM). Losses are compensated by fibre amplifiers (EDFA). The temporal pulse evolution in the loops can be mapped onto 1+1D mesh-lattices spanned by the discrete time *m* and position *n*. In contrast to the passive lattice (**b**) a constant gain (red) in the long loop and loss (blue) in the short loop are equivalent to amplified and attenuated diagonal paths through the lattice (**c**). (**d**) By alternating gain and loss on every other round trip (purple) and by inserting an appropriate phase modulation a PT-symmetric system can be generated, which consists of amplifying and lossy waveguides.

Our experimental platform consists of two coupled fibre loops having slightly different lengths^11^,^50^,^51^,^52^ (see Supplementary Fig. 1 and Supplementary Methods). Like in time multiplexing, subsequent passes through the short and the long loop cause a pulse to spread on a time mesh lattice with discrete arrival times being equivalent to positions in the spatial domain (see Fig. 1). As group velocity dispersion is negligible in our setup, each pulse is completely characterized by a single complex amplitude that is denoted by 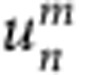 and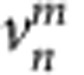 for the short and the long loop, respectively. Here *m* stands for the time interval as measured in round trips and *n* denotes the position of a single pulse during one cycle. When a pulse travels through the longer loop, it will not only step in time from *m* to *m*+1, but will also be slightly delayed thus hopping from position *n* to *n*+1. Conversely, the propagation in the short loop is equivalent to shifting the pulse to the descendent position at *n*−1. In what follows, we discuss the ensuing optical evolution in a co-moving reference frame.

During each passage through a loop, pulses accumulate nonlinear phase shifts 
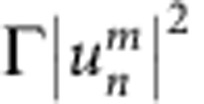
 and 
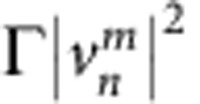
, respectively, where Γ represents the effective nonlinearity of the system^32^. All unwanted losses are compensated by using erbium-doped fibre amplifiers. Fine tuning of amplitude modulations is obtained by acousto-optic modulators (AOM) resulting in an effective loss or gain factor of *G*_*u*,*v*_ for the short and the long loop. A phase modulator (PM) inserted in the short loop also controls the phases of the pulses by inducing an arbitrary phase potential^11^,^27^ *ϕ*_*n*_. The overall dynamics of this system are described by^52^:


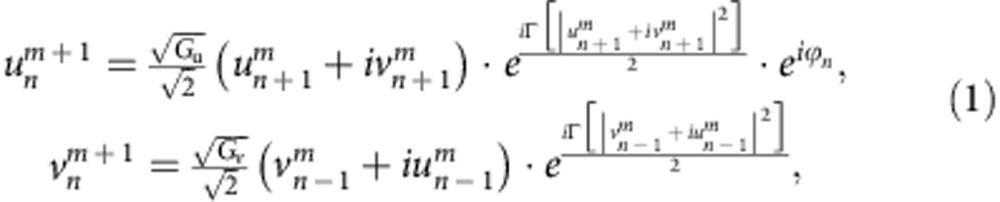


where the respective amplitudes are determined right behind the 50% coupler that connects both loops (see Supplementary Note 1 and Supplementary Fig. 2). Equation (1) are used to model our system in all forthcoming simulations. As it will be shown later, by rearranging the parameters in this versatile-loop system, one can synthesize three general types of the mesh lattices: conservative Hermitian lattices (Fig. 1b), lattices with local PT symmetry (Fig. 1c) and globally PT-symmetric lattices (Fig. 1d). In what follows, we investigate soliton dynamics in all these three cases.

### Soliton formation in Hermitian mesh lattices

Before investigating solitons in PT-symmetric lattices, it is beneficial to explore this possibility in the corresponding conservative environment. In general, the properties of lattice solitary waves critically depend on the band structure of the associated linear system (see Supplementary Fig. 3 and Supplementary Note 2). In the absence of any gain/loss and phase modulation (*G*_*u,v*_=1, *ϕ*_*n*_=0, Γ=0), a substitution of a Floquet–Bloch ansatz


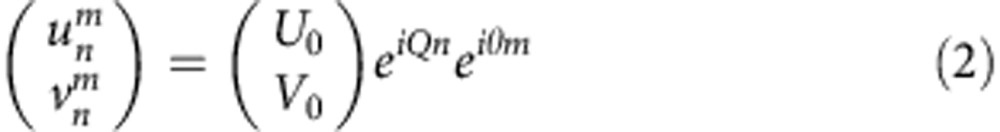


in equation (1) leads to the dispersion relation.


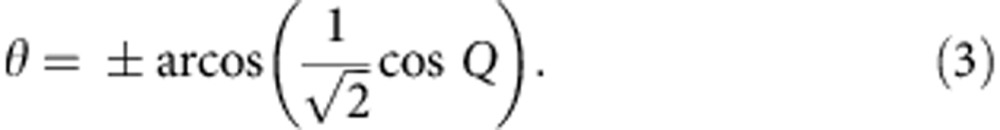


**Figure 2:**
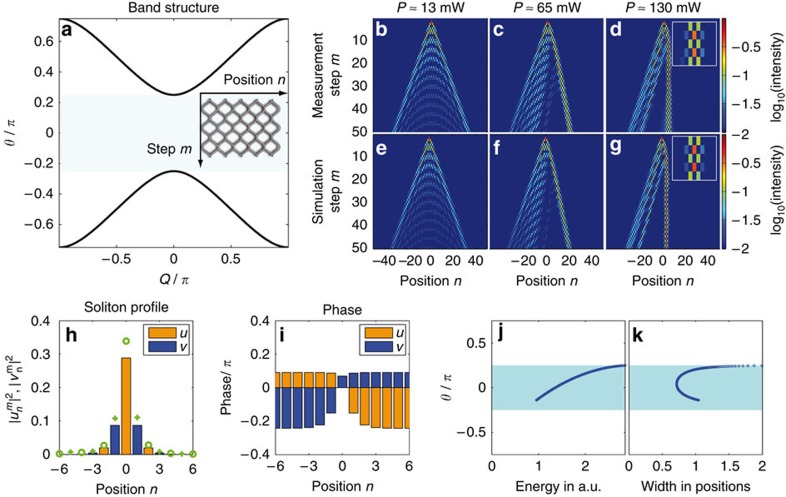
Formation of the double-discrete soliton on a passive lattice. (**a**) The band structure is split into two bands, separated by a gap of width *π*/2. The excitation of a single-lattice site in the long loop initiates a light walk for linear power levels *P*≈13 mW (**b**,**e**). If the input power is increased *P*≈65 mW (**c**,**f**), one of the two branches bends towards the center and repels the remaining light. At high powers *P*≈130 mW (**d**,**g**) a soliton is formed, which is dominated by a single pulse, which switches between loops. The insets at *P*≈130 mW show the temporal dynamics over five time steps around *m*=25. Only pulses propagating in the short loop are shown. (**h**) Comparison between a numerically (bars) and an experimentally (markers) determined soliton profile in the longer (*v*, cross) and in the shorter (*u*, circle) loop. (**i**), numerically determined phase distribution along the exact soliton profile. The values for the propagation constant of the soliton are lying inside the band gap. From the lower to the upper edge of the band gap the total energy *E* monotonically increases (**j**). At the edge of the band gap the width *w* of the soliton tends to infinity, while at the center of the gap the soliton has a minimum width <1 position (**k**). (**j**,**k**) are based on numerically determined soliton solutions.

Here θ and *Q* stand for the longitudinal propagation constant and the transverse Bloch momentum respectively. The band structure depicted in Fig. 2a consists of two bands, which are separated by a gap of *π*/2. Due to different dispersion characteristics, the Kerr nonlinearity^32^ of the fibre has a focusing effect on the field distribution populating the upper band, and a defocusing one on pulses in the lower band^52^,^62^. By injecting a single low-intensity pulse at one lattice site, all states of the band structure are excited simultaneously (see Fig. 2b,e). In this linear regime, the field spreads ballistically^34^ between two intensity lobes formed by waves having zero group velocity dispersion (Fig. 2a). As we will show, this so-called classical light walk is considerably modified in the presence of nonlinearity.

By increasing the pulse power, for an initial excitation of the long/short loop, energy accumulates in the slow/fast branch, which then loses mobility. As a result, the dominant part of this energy distribution bends towards the center and repels remaining pulses (see Fig. 2c,f). For higher input powers, a quasi-stationary soliton state forms. It consists of a strong pulse that alternates between both loops, thus staying at rest in the co-moving frame of reference (see Fig. 2d,g). This strong pulse is accompanied by other much weaker ones, which also switch between the two loops in a countercyclical manner with respect to the strong pulse. Despite this unusual dynamics, the observed double-discrete soliton still represents a stationary nonlinear state that resides primarily within an elementary cell of this mesh lattice. Numerical results corroborating this observed behaviour are provided in Fig. 2e–g and in Supplementary Fig. 4.

To theoretically analyse this double-discrete soliton, we assume a stationary profile (see Supplementary Fig. 5 and Supplementary Note 3)


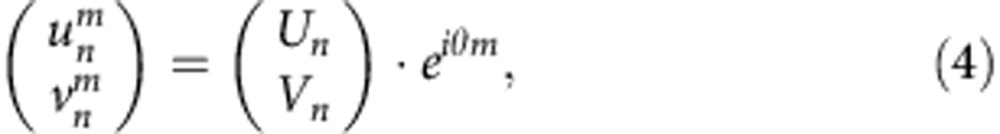


and use a nonlinear mode solver to determine the respective amplitudes *U*_*n*_ and *V*_*n*_ as a function of the propagation constant θ. A particular (*U*_*n*_,*V*_*n*_)^*T*^ soliton solution along with its associated phase profile is depicted in Fig. 2h,i. Here we display a highly localized wave, similar to the one observed in the experiment.

The variation of the soliton propagation constant (eigenvalue *θ*), as a function of its total energy *E*=∑_*n*_(|*U*_*n*_|^2^+|*V*_*n*_|^2^) is depicted in Fig. 2j. Similarly Fig. 2k depicts the eigenvalue–soliton width curve, which is determined by the centred second moment of the field distribution. According to these figures, as the power increases, the soliton eigenvalue separates from the top of the band and enters the band gap. The soliton contracts to less than one lattice spacing in the center of the band gap, but due to enhanced coupling to linear modes the width of the soliton diverges close to both band edges. Although the soliton represents a stationary state, its internal dynamics are characterized by two coupled, but counteracting energy flows in the two loops corresponding to the two opposite phase gradients in the soliton profile (see Fig. 2i). By further increasing the power, the soliton eigenvalue increases until reaching the upper band where this localized state disappears again.

### Solitons in lattices with local PT symmetry

We next consider soliton formation in dissipative mesh lattices. We first introduce losses in only one loop *G*_*u*_=1/*G*, and gain *G*_*v*_=*G* in the other thus resulting in the lattice depicted in Fig. 1c. In general, a discrete mesh lattice satisfies PT symmetry provided that it remains invariant under *n*→−*n*, *m*→−*m* and after complex conjugation (exchanging gain with loss, see Supplementary Fig. 6). In this respect, the lattice is Fig. 1c is not a genuine PT-symmetric one. Instead, this lattice is locally PT-symmetric, meaning that only along any cross section *m*=*m*_0_ the resulting lattice is invariant under *n*→−*n* and after gain and loss reversal. Therefore, this system, hereby defined as locally PT-symmetric fulfills the conditions for P and T symmetry only at every time step.

By adopting the same ansatz used in equation (2), it follows that the band structure of the corresponding linear system (for an infinite lattice) is given by the modified dispersion relation (see Supplementary Note 2 and Supplementary Fig. 3):


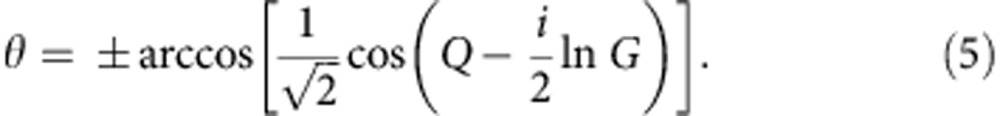


**Figure 3:**
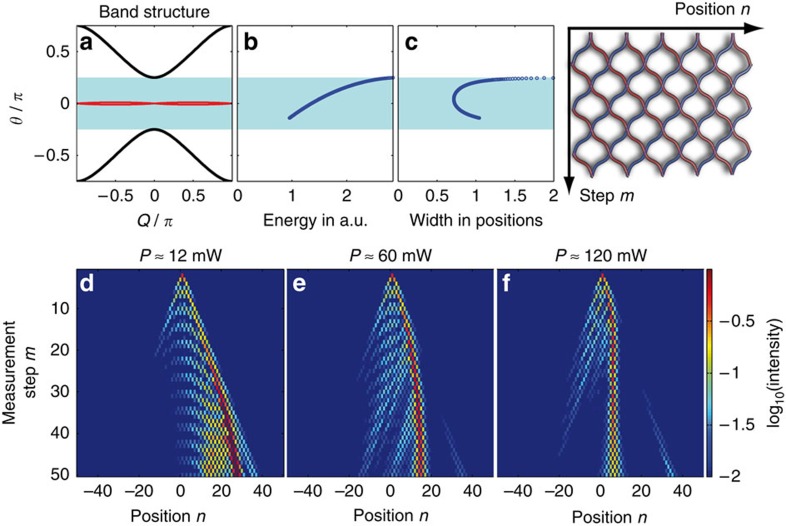
Formation of double-discrete solitons in locally PT-symmetric lattices (measurement). (**a**) The band structure of the infinite lattice with *G*=1.1 is complex over the Brillouin zone. The real and imaginary parts are shown in black and red, respectively. (**b**,**c**) The eigenvalue–energy and width parametric curves of the discrete solitons in this lattice. As the power gradually increases (**d**–**f**) a discrete soliton forms at *P*≈120 mW.

Note, that the eigenvalue *θ* is now complex over nearly the entire range of the Bloch momentum *Q* (Fig. 3a). However, numerical analysis show, that for a finite configuration, the eigenvalues remain real below a certain critical value of gain, as it is usually the case for PT-symmetric systems. Due to experimental restrictions, we investigate a large but finite system.

To ensure that the power is always bounded in the system, the lattice must be finite with respect to *n*. In our experiment, such boundary condition is imposed by having the acousto-optic modulators inducing high attenuation at the outermost positions *n*±*N*, thus causing the pulse amplitudes to vanish at the edges (
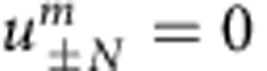
 and 
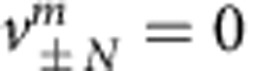
).

Quite interestingly, in this locally PT-symmetric environment, stable discrete solitons exist (see Supplementary Note 4 and Supplementary Figs. 7–9). If the initial distribution does not match the soliton profile, the pulse travels towards higher *n* and is amplified during its propagation until it becomes locked to a single position due to nonlinearly induced self-localization. Once the newly formed discrete soliton stays on one lattice site, the pulse permanently alternates between both loops and is on average neither amplified nor attenuated (see Fig. 3d–f and Supplementary Fig. 10). This process happens to be independent of the lattice size and thus stable solitons even exist on the infinite and unstable lattice. Due to the high symmetry of the mesh lattice involved, the only difference between injecting the initial distribution in the long or short loop is a negligible initial amplification or attenuation.

In general, dissipative solitons appear in many areas of optics^35^,^53^,^54^,^55^,^56^,^57^. As opposed to conservative ones, which manifest themselves as a continuous parametric family^32^, dissipative solitons are completely determined by the parameters of the system and hence exist as fixed point solutions^35^. Surprisingly, in our case, the locally PT-symmetric lattices allow again these dissipative discrete solitons to form a continuous branch (see Fig. 3b,c), very much like their conservative counterparts. The existence of a continuous branch of solutions in PT-symmetric arrangements can be intuitively understood on the basis that both systems exhibit entire real spectra in their linear regime. This close relationship between these two configurations leads in both cases to the presence of a parametric family of soliton waves. It is, however, worth mentioning that these solitons behave like attractors and hence a broad Gaussian distribution tends to form narrow fundamental self-trapped states—a process observed in our experiments (see the Supplementary Fig. 9).

### PT-symmetric lattice solitons

In this section we report the observation of optical self-localized nonlinear states in globally PT-symmetric lattices. While a number of PT-symmetric arrangements have already been theoretically investigated in both the linear and nonlinear regime, their nonlinear soliton response still remains experimentally unexplored. In optics, the respective potential is defined by the complex refractive index distribution and has to satisfy the PT-symmetric conditions to allow for real eigenvalues^8^,^63^,^64^,^65^. Again, while the real part of the potential has to be symmetric, the imaginary part must be antisymmetric with respect to the spatial coordinate (see Supplementary Fig. 6). As indicated above, this condition is necessary but not sufficient^8^. For example, the potential of the periodic system introduced in the previous section has an antisymmetric imaginary part and a vanishing real component (see Fig. 3), but still has no real eigenvalues for *G*≠1, if the system size tends to infinity (equation 5). On the other hand, this potential can be readily modified to establish a PT-symmetric periodic environment with a finite threshold. This can be achieved by exchanging the amplifying and lossy paths after every two round trips (Fig. 1d) and by imposing a phase potential according to


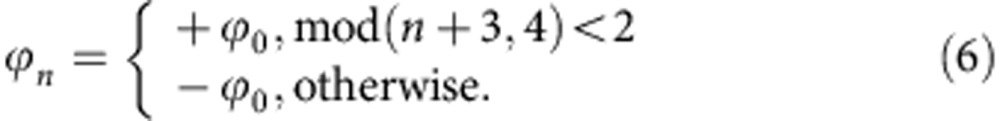


In this case, the allowed bands and gaps can be obtained by using a Floquet–Bloch ansatz 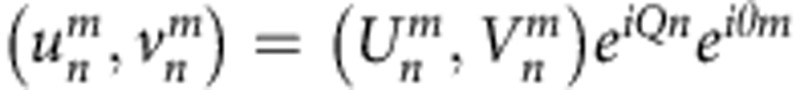 where 
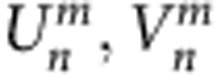
 represent periodic discrete Bloch wave functions with periodicities of *n*_0_=4 and *m*_0_=2 in *n* and *m* repesctively. The band structure of this PT-symmetric lattice is given by (see Supplementary Note 2 and Supplementary Fig. 3f):


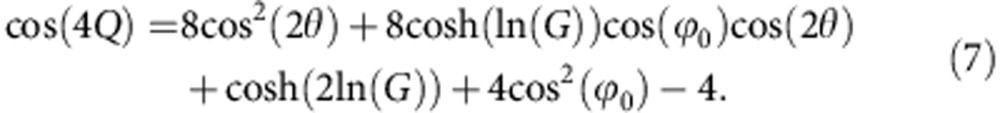


**Figure 4:**
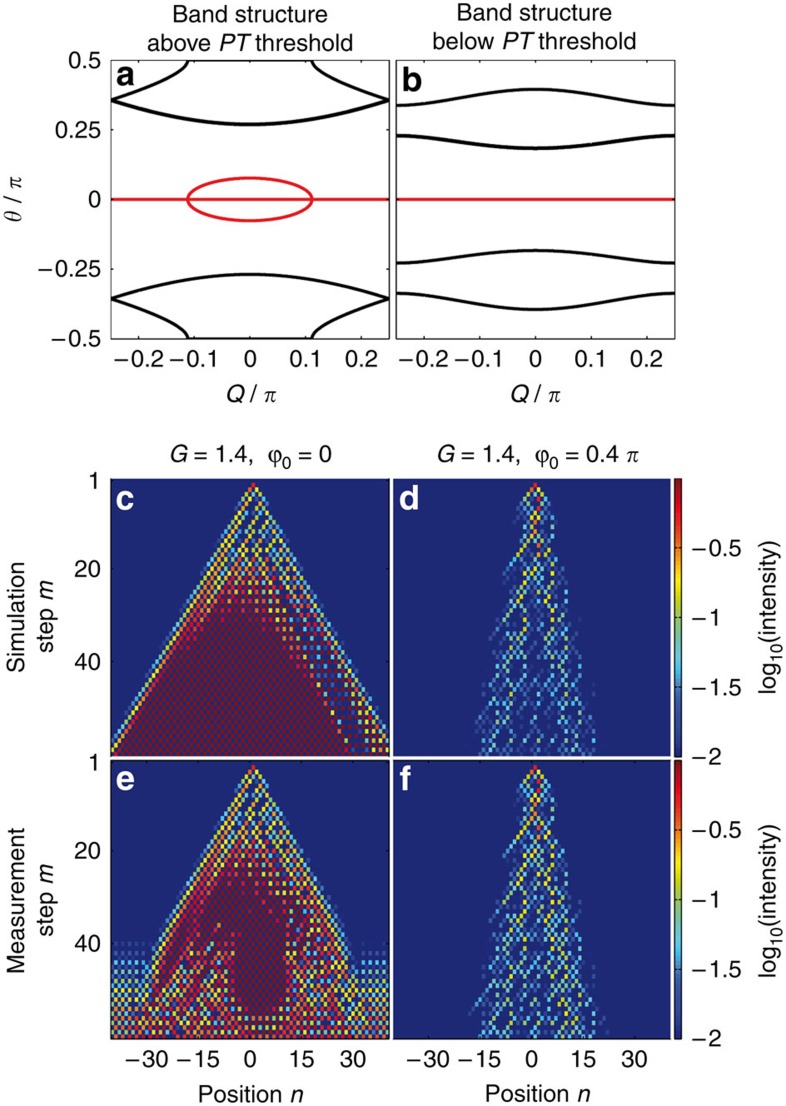
Linear propagation in a PT-symmetric lattice. The band structure (**a**) above (*G*=1.4,*ϕ*_0_=0) and (**b**) below (*G*=1.4,*ϕ*_0_=0.4*π*) the PT threshold (real part in black and imaginary part in red). When a single position is excited the wavepacket spreading occurs that either experiences amplification in the broken regime (**c**,**e**) or remains neutral below threshold (**d**,**f**). The power is ∼13 mW.

Figure 4a,b depict this dispersion relation for two different values of (*ϕ*_0_,*G*). Evidently, in this configuration, the eigenvalues *θ* are completely real in certain regions of the parameter map spanned by *ϕ*_0_ and *G* (below PT threshold), and become complex above the PT-symmetry-breaking threshold^11^,^51^ (see Supplementary Fig. 11).

Before exploring soliton evolution in this PT array, we first consider its linear response (see Supplementary Fig. 12). If *G*=1.4 and *ϕ*_0_=0, the system is in the broken PT symmetry regime (see band structure of Fig. 4a) and as a result the energy not only disperses but also grows exponentially with distance (Fig. 4c,e). Due to the saturation of our detectors and erbium-doped amplifiers, the exponential behaviour in the PT-broken case can only be observed in good agreement with simulations for the first 30 time steps while the saturation effects dominate afterwards (see Supplementary Fig. 13) On the other hand, if *G*=1.4 and *ϕ*_0_=0.4*π*, then PT symmetry is restored and the bands become entirely real (Fig. 4b). In this case the energy remains bounded while at the same time a PT light walk^47^ takes place leading to broadening effects (Fig. 4d,f).

**Figure 5:**
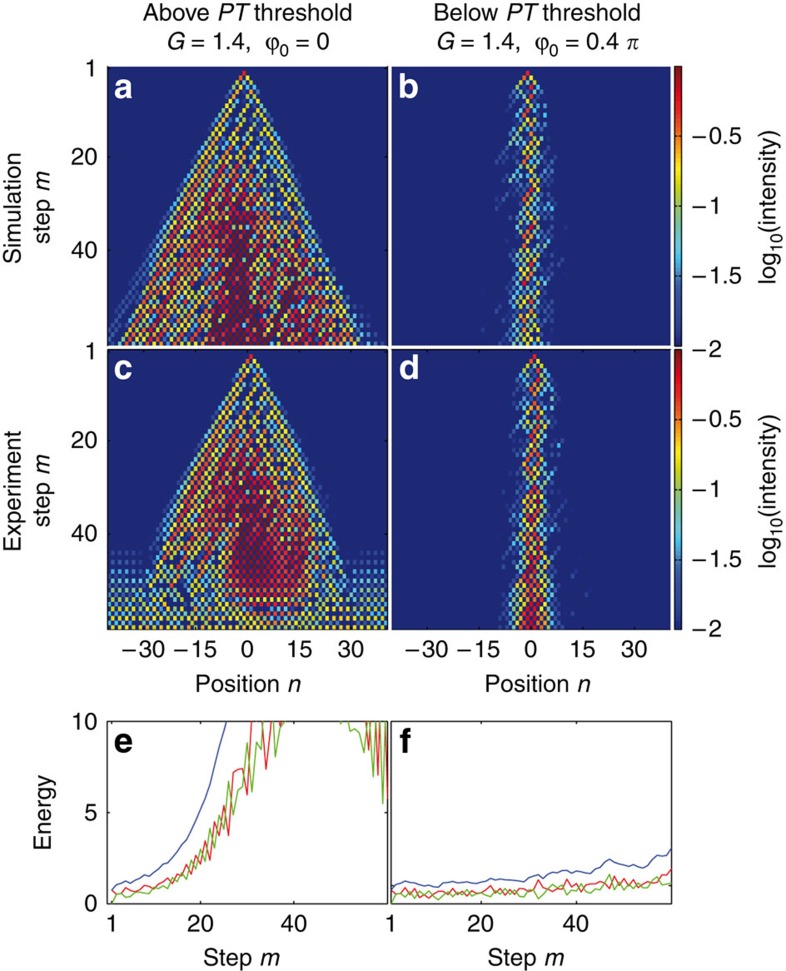
Nonlinear self-trapping in a PT-symmetric mesh lattice. Same as in Fig. 4 but in the nonlinear regime. The nonlinearity cannot prevent the expansion above the PT threshold (**a**,**c**,**e**). Yet at high powers a discrete soliton is formed once PT symmetry is restored (*G*=1.4,*ϕ*_0_=0.4*π*) (**b**,**d**,**f**). In this case internal oscillations are observed because of mode interference effects. For simulations a nonlinear factor of Γ=0.24*π* was chosen. In the experiment the power of the initial pulse is *P*_*in*_≈40 mW.

Once the power of the single initial pulse is raised, nonlinear processes come into play and hence discrete solitons can in principle form in this PT configuration. In general, the strong phase potential slightly decouples the adjacent lattice sites of the mesh structure which in turn hinders the spreading of the initial distribution. As a result, nonlinear effects can be observed at much lower peak powers (see Supplementary Fig. 14). When the associated linear lattice is operated above the critical threshold the system becomes unstable and the nonlinearity is unable to establish a solitary wave (Fig. 5a,c). This exponential increase in energy is shown in Fig. 5e. Conversely, when the band structure is real (below PT threshold), an optical soliton is observed as depicted in Fig. 5b,d. As the PT lattice has a four-side spatial periodicity a single-site excitation does not result in a stationary profile but instead excites strong internal oscillations around the PT soliton. In all cases the soliton remains almost invariant during propagation without any appreciable increase in its energy (Fig. 5f).

**Figure 6:**
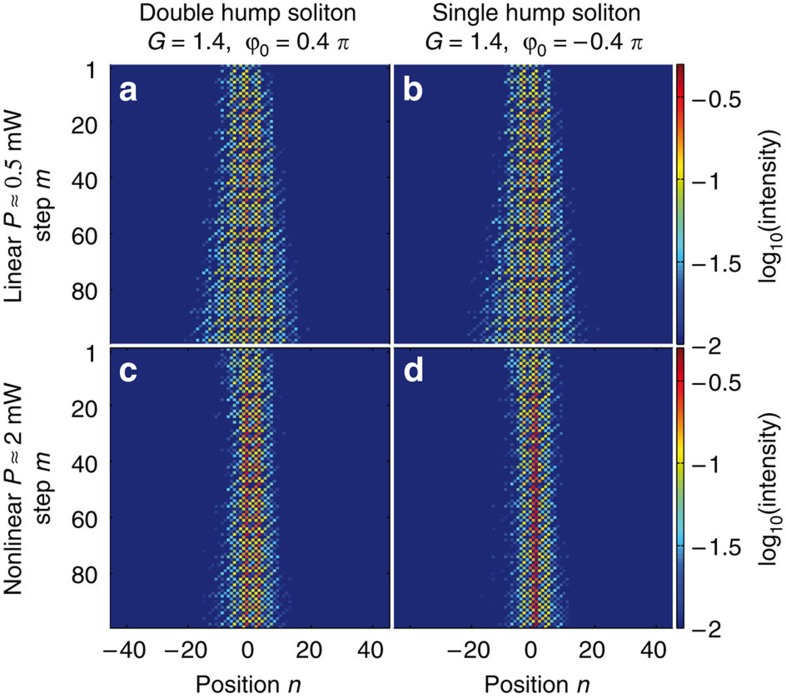
Formation of a broad PT soliton. A broad Gaussian distribution populating the focusing band is excited. In the linear regime (**a**,**b**) the beam spreads, while in the nonlinear regime a broad double hump (**c**) or single hump (**d**) PT soliton forms which can propagate for 100 round trips. Note that for observing the single and double hump solitons the sign of the phase potential is switched between *ϕ*_0_=−0.4*π* and *ϕ*_0_=+0.4*π*, with *G*=1.4 for all cases. The estimated peak power in the experiment is about 2 mW for the nonlinear regime and below 0.5 mW for the linear regime.

To launch wider solitons, a broad Gaussian distribution with a flat phase front is used to excite the system. In this case, because of nonlinearity, Schrödinger-like solitons^29^ form in the PT lattice (see Fig. 6 and Supplementary Figs 15 and 16), which are similar in shape and behaviour to solitons arising from the nonlinear Schrödinger equation. Note that, if the above mentioned phase potential (equation 6) is generated without any amplification or attenuation, the system behaves like a bi-periodic waveguide array where transport is considerably suppressed. In the nonlinear regime, discrete solitons are formed that happen to be quite immobile in a way similar to those found in waveguide arrays^31^ (see Supplementary Fig. 16). In the presence of PT-symmetric gain/loss, the intensity profile of the soliton is close to that of a conservative self-trapped wave and is critically determined by the phase potential. As in the previous case, these PT lattice solitons belong to a continuous parametric family of solutions. As numerical simulations indicate, mainly its phase profile adapts to gain and loss, while their interaction is still similar to that of conservative solitons (see Supplementary Fig. 17 and Supplementary Note 5). Although the observed PT solitons are stable for the complete experimental range of 100 round trips, simulations suggest that an extremely weak intrinsic instability is present due to a small imaginary part (because of nonlinearity) in the propagation constant. This instability is caused by the physical separation between amplification/attenuation and nonlinear propagation in our setup. In a sense, our mesh lattice platform can be viewed as an experimental implementation of the numerical split-step method when solving the nonlinear Schrödinger equation where errors arise due to discretization.

### PT-symmetric saturable absorber

**Figure 7:**
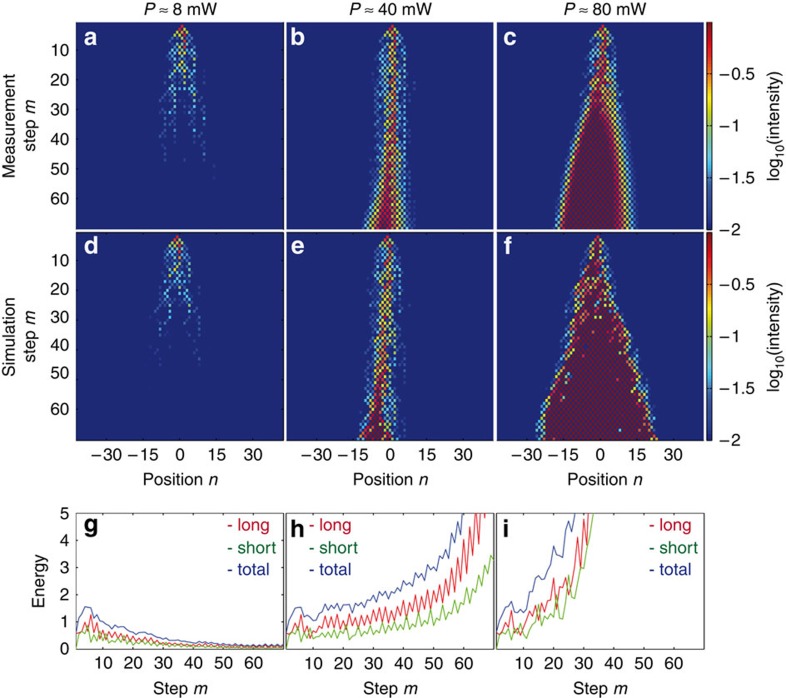
Synthesizing a saturable absorber. A single-lattice site is excited in a PT-symmetric lattice with *ϕ*_0_=0.4*π*, *G*=2 involving a global loss of 5%. In the linear regime the attenuation filters out all low-level noise signals (**a**,**d**). If the power is increased (**b**,**e**), the nonlinear mode starts to localize and experiences a lower loss during propagation by choosing the best path (**c**,**f**). At high powers losses can even be avoided and gain can be globally reached. (**g**–**i**) shows the energy from the measured data in both loops (blue), in the long loop (green) and in the short loop (red). While in the experiment the power of the initial pulse is varied, in the simulation nonlinear factors of Γ=0, Γ=0.26*π* and Γ=0.52*π* were assumed for a best fit between simulation and experiment.

Finally, we discuss the possibility of using such PT synthetic nonlinear lattices as a new class of saturable absorbers^63^. This is achieved by adding a global loss to an otherwise PT-symmetric nonlinear mesh lattice. When a low-intensity pulse is injected into this system, it spreads linearly while all modes decay due to the added loss (Fig. 7a,d). As a result after several round-trips the total energy of the system vanishes. On the other hand, by increasing the power of the injected pulse, a self-trapped wavepacket forms which experiences much lower losses compared with a linearly diffracting wave (Fig. 7b,e). By further increasing the power of the input pulse, this localized wave can even amplify itself (Fig. 7c,f). Such a behaviour of nonlinear waves^64^,^65^,^66^ is strongly related to saturable absorbers that are widely used in Q-switched laser cavities and in ultra-short optical pulse arrangements^67^,^68^,^69^.

## Discussion

In conclusion, we have experimentally demonstrated stable optical discrete solitons in PT-symmetric mesh lattices. We have shown that this class of self-trapped states is possible in either locally or globally PT-symmetric systems. The existence curves of this continuous parametric family of local PT-symmetric soliton solutions were determined and their stability properties were also investigated. The possibility of realizing a new class of saturable absorbers based on such arrangements was considered. Our experimental platform can provide a versatile test bed to explore a wide range of phenomena and processes in nonlinear and non-Hermitian environments.

## Methods

### Numerical soliton solver

To theoretically analyse solitons in complex lattices a nonlinear mode solver was used based on Newton's method. For a propagation constant *θ*=−0.2*π*, an initial Gaussian distribution with a width of five positions and an amplitude of 0.2 was assumed. While the propagation constant was kept fixed, the real and imaginary parts of the spatial distribution were varied until a solution was obtained. After each iteration, the residual error was estimated and used as a measure of convergence. Distributions with a residual error below or equal to 10^−16^ were assumed to be a stationary solution of the evolution equations. Starting from a solution close to the band gap at *θ*=−0.2*π*, all other soliton solutions were pursued by assuming the last solution as the starting point. On lattices with gain and loss, the solver was initiated with conservative solutions.

### Signal processing and reproducibility

The electronic signals of the photodiodes were first amplified and afterwards recorded by an oscilloscope. An internal averaging function of the oscilloscope was used to estimate the mean value out of about 40 realizations. While all experiments feature a high reproducibility, the polarization and the gain of the erbium-doped fibre amplifiers has to be readjusted about every 10 min to compensate for long term drifts. For a high fidelity of the acquired data, the setup was not altered during a sweep over different initial powers or between the measurement of the conservative system and the global PT-symmetric system. Therefore, a direct comparison between the cases with and without amplification or attenuation was made possible.

A detailed description of the experimental methods is provided in the Supplementary Methods section.

## Additional information

**How to cite this article:** Wimmer, M. *et al.* Observation of optical solitons in PT-symmetric lattices. *Nat. Commun.* 6:7782 doi: 10.1038/ncomms8782 (2015).

## Supplementary Material

Supplementary Information Supplementary Figures 1-17, Supplementary Note 1-5, Supplementary Methods and Supplementary References.
