# Supplementary material for: Observation of optical solitons in PT-symmetric lattices
Source: Nat Commun. 2015 Jul 28;6:7782. doi: 10.1038/ncomms8782 (PMC4525206; doi:10.1038/ncomms8782)
Supplement: Supplementary Information — Supplementary Figures 1-17, Supplementary Note 1-5, Supplementary Methods and Supplementary References. [file ncomms8782-s1.pdf]

Supplementary Figures

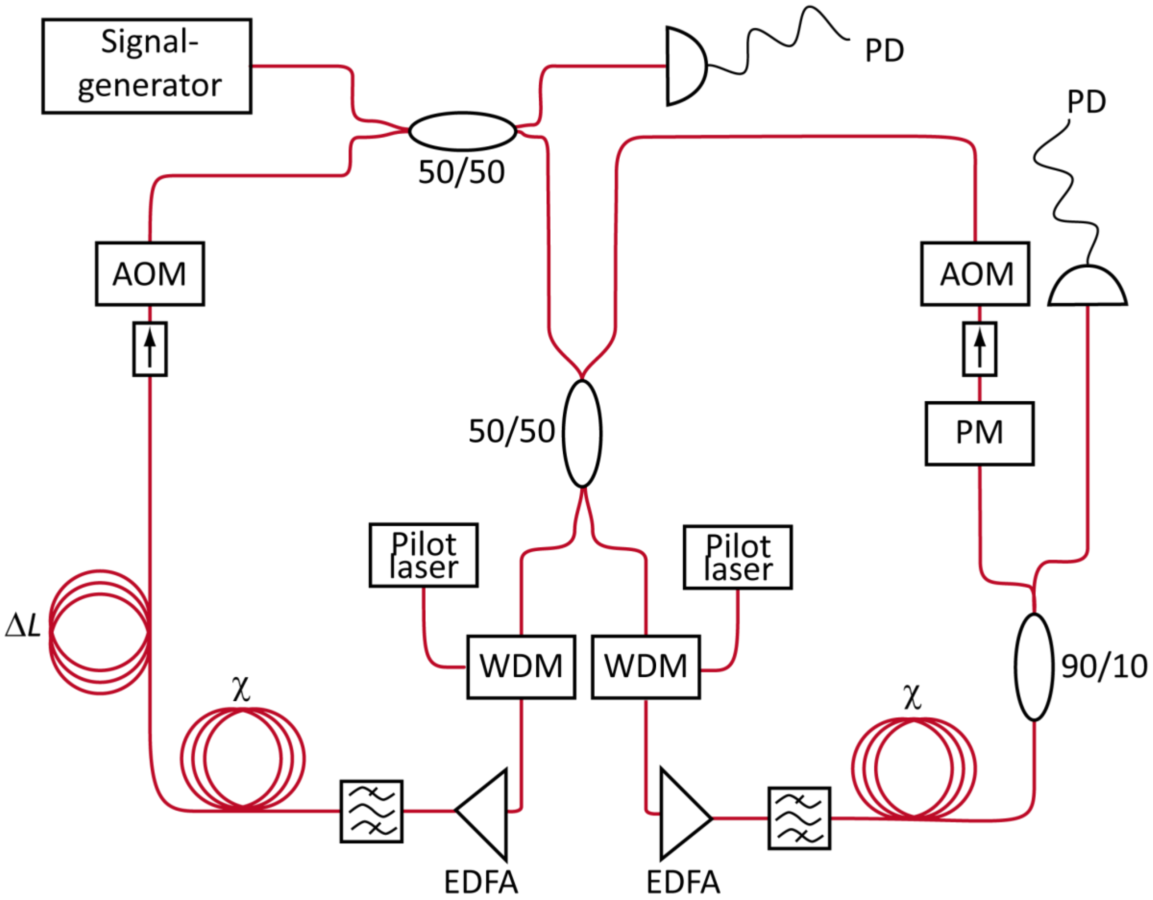

Supplementary Figure 1 | Detailed scheme of the nonlinear fiber loops. Pulses circulate in two optical fiber loops, which are connected by a central 50/50 coupler. All components are fiber-coupled. PD: Photodiode; AOM: Acousto-optic modulator; PM: Phase modulator; WDM: Wavelength-division multiplexing coupler; EDFA: Erbium-doped fiber amplifier; DCF: Dispersion-compensating fiber; χ: total nonlinearity coefficient; ISO: Optical isolator. Figure adapted from Ref. [4].

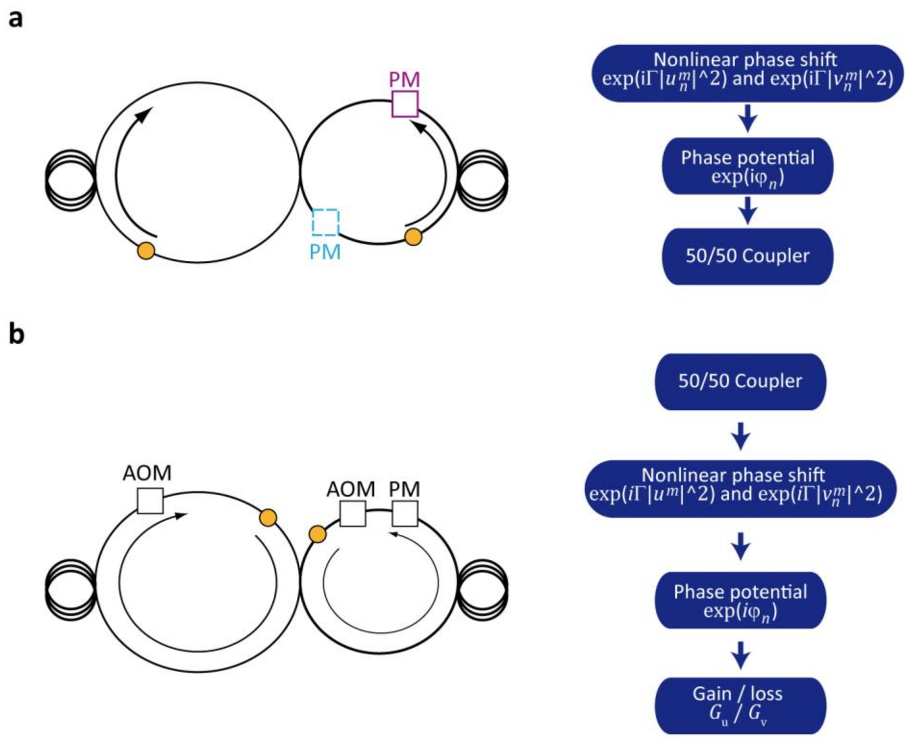

Supplementary Figure 2 | Setting up evolution equations for optical mesh lattices. **a**, In the absence of gain and loss, the evolution is governed by a nonlinear phase shift acquired during the propagation through a DCF and a linear phase potential exerted through phase modulators. The position of these phase modulators is irrelevant since all components commute with each other. **b**, In case of an active amplitude modulation of the pulses, the ordering of the components becomes important, since the two processes of amplification/attenuation and the nonlinear phase shift do not commute. In experiment, the pulses are first split up at the central 50/50 coupler and then experience a nonlinear phase shift. At the end of a single round trip, amplitudes and phases of the pulses are modulated.

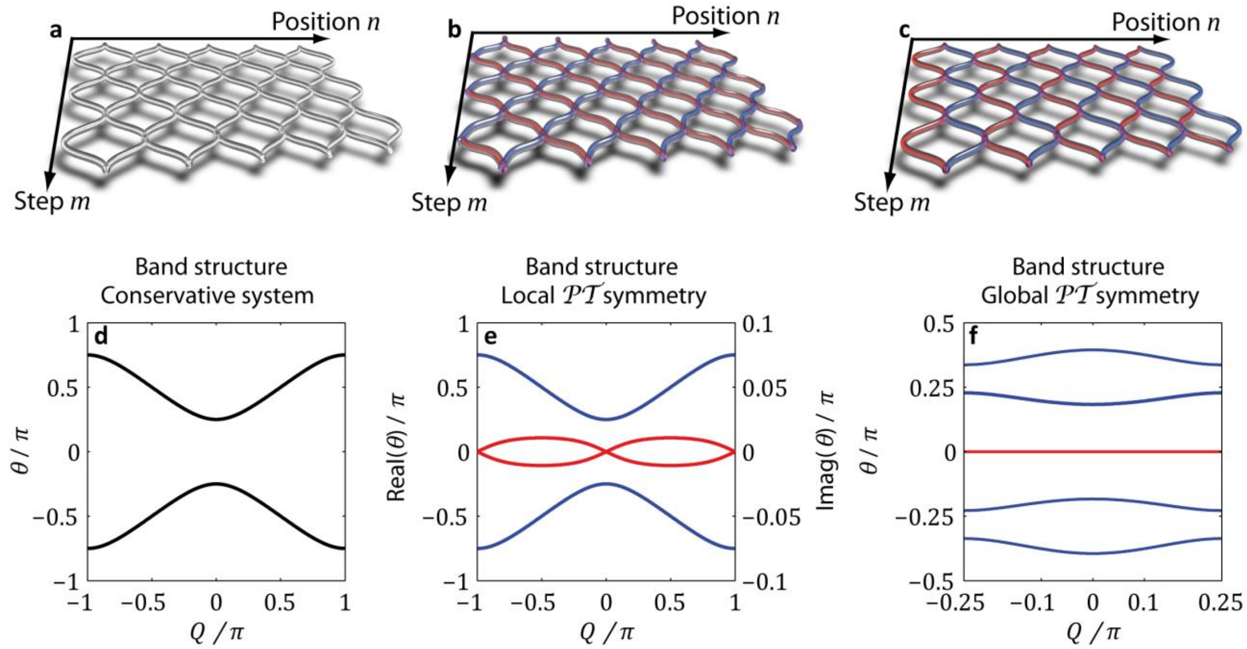

Supplementary Figure 3 | Comparison of the mesh lattices and their associated band structures. In all investigated cases light spreads on a 1+1D lattice spanned by a discrete time m and a discrete position n (**a**-**c**). If there is no amplification and attenuation involved (**a**) the band structure is completely real and split up into two bands (**d**). In case of the local $\mathcal{PT}$-symmetric lattice (**b**) all pulses traveling to the right are amplified while pulses moving into the opposite direction are attenuated. The corresponding band structure (**e**) has a nonvanishing imaginary part (red), which is magnified by a factor of 10 with respect to the real part (blue) for better visualization. **c**, By alternating gain and loss every roundtrip single waveguides with gain and loss are generated. The increased size of the unit cell leads to an additional splitting up into four disjoint bands with a vanishing imaginary part below the $\mathcal{PT}$ threshold.

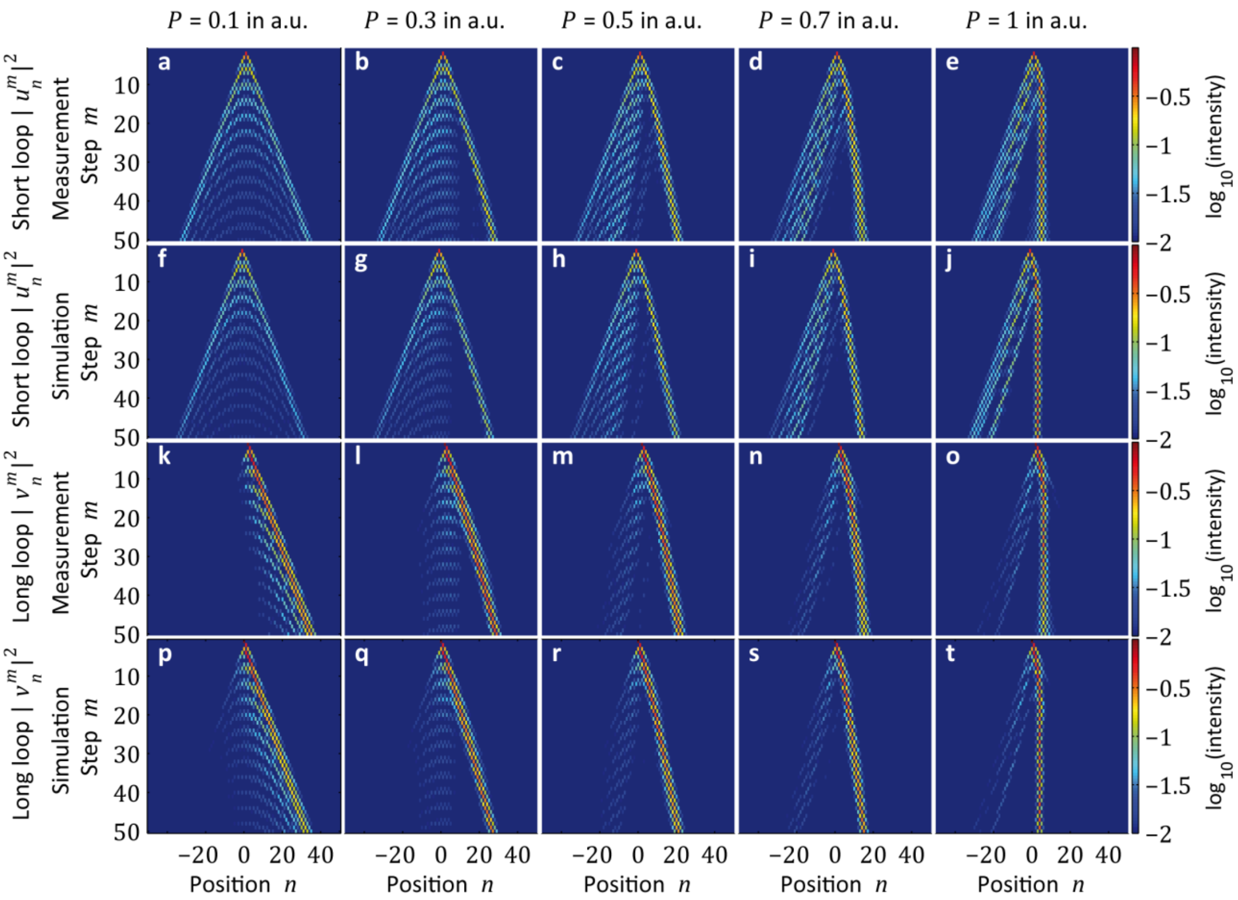

Supplementary Figure 4 | Simulations and measurements of the light walk on the conservative lattice. Starting with the excitation of a single lattice point in one of the loops, a so-called light walk is initiated (first column) spanned by two fast moving side arms propagating with the maximum group velocity but having different power. An increase of the initial power leads to a bending of the stronger right sidearm towards the center, which repels the remaining pulse distribution from the center to the left side (second to fourth column). At high powers, this sidearm forms a stable soliton, which alternates between both loops and thus stays at rest in the commoving frame of reference (fifth column). In the simulation, a maximum nonlinear factor of $\Gamma \approx 0.84\pi$ was assumed for a best fit to the measurement and scaled linearly with the input power.

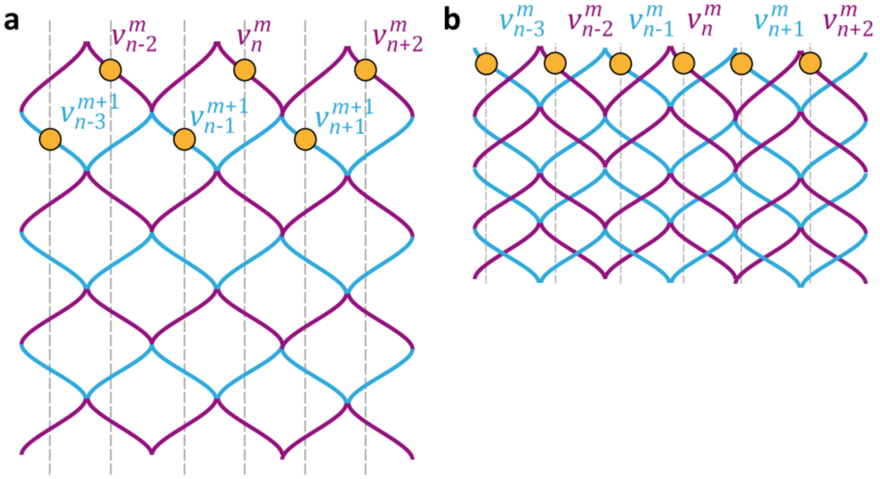

Supplementary Figure 5 | Reduction of the double step evolution to a single step dynamic. a, On the mesh lattice as realized in the experiment only every second position can be reached, since pulses must either increase or decrease their position by 1 during one time step thus constantly switching between even and odd positions. **b,** To simplify the numerical search for soliton profiles, every second row is moved upwards. In this case, every odd position stands for the dynamic of the first time step and every even position for the second time step. Therefore, two time steps with sparse occupation are reduced to a single time step with a dense occupation of the lattice.

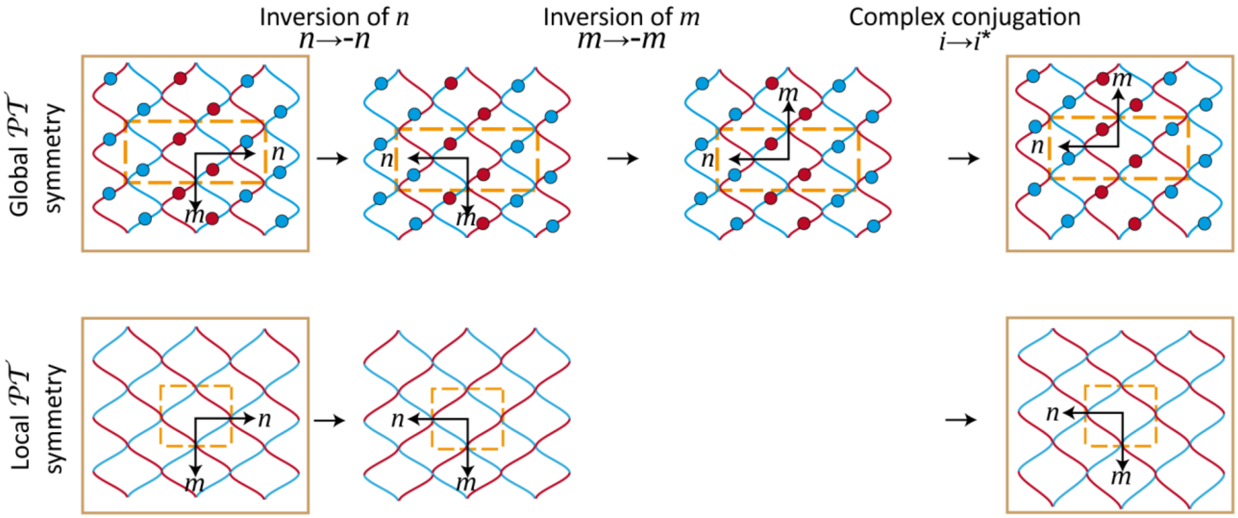

Supplementary Figure 6 | Underlying symmetry operations for global and local $\mathcal{PT}$-symmetric lattices. The global $\mathcal{PT}$-symmetric mesh lattice is fully restored after inversion of space ($m \to -m, n \to -n$) and complex conjugation (gain ↔ loss) operations. This kind of symmetry is also present if m is regarded as the evolution variable attributed to an effective time. However, when gain (red) and loss (blue) waveguides are exchanged in every time step, the resulting mesh lattice is called to be locally $\mathcal{PT}$-symmetric in contrast to the globally $\mathcal{PT}$-symmetric system. In this latter case, the lattice is $\mathcal{PT}$-symmetric only locally, meaning that only along any cross section $m = m_0$ the resulting lattice is invariant under $n \to -n$ and after complex conjugation. Note that the global $\mathcal{PT}$-symmetric lattice involves a phase potential (red and blue circles) which leads to an increased unit cell compared to the local $\mathcal{PT}$-symmetric case (in each case the unit cell is shown with the dashed orange box).

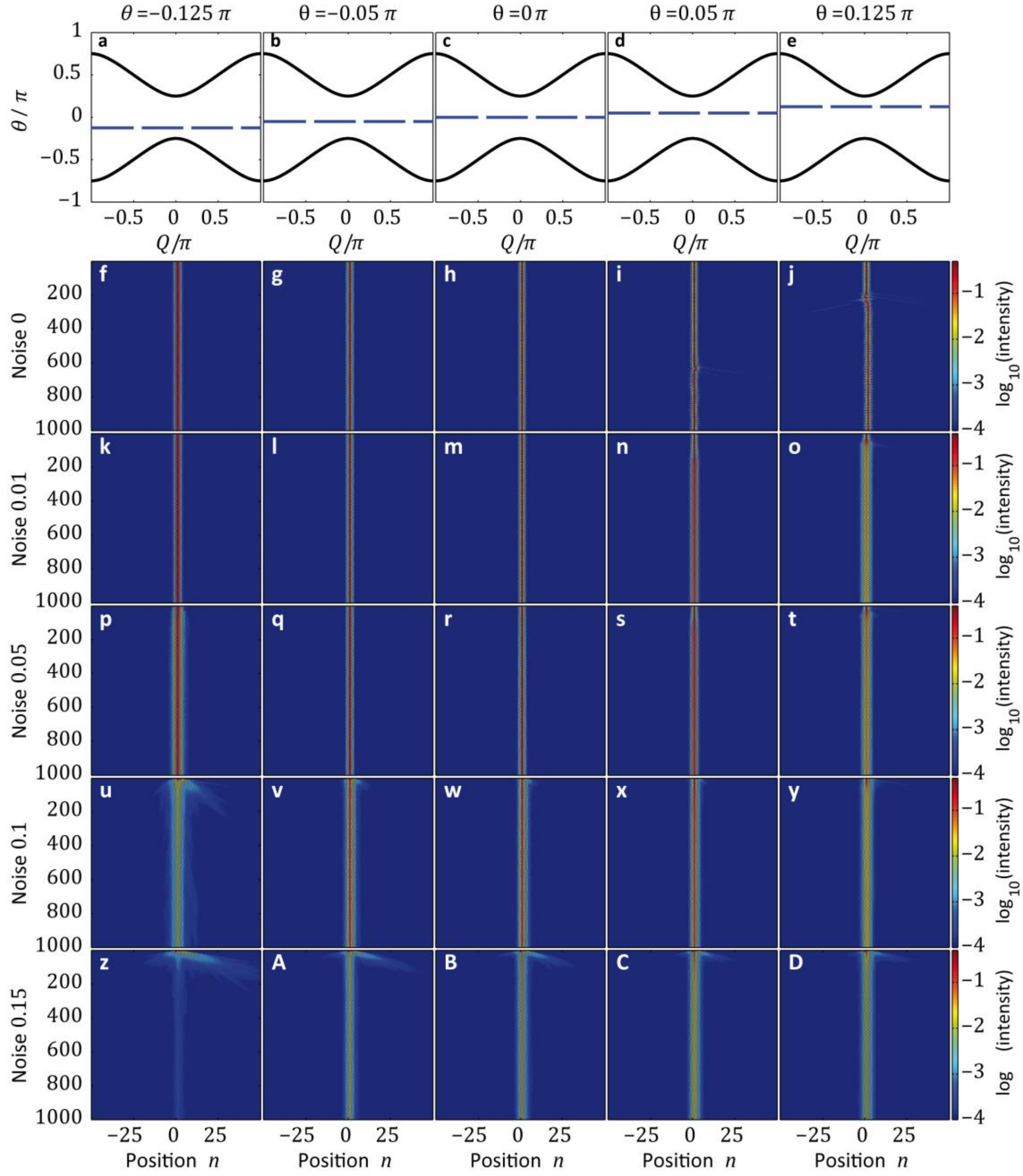

Supplementary Figure 7 | Qualitative stability analysis of the conservative solitons. Numerically determined soliton profiles are perturbed by an equally distributed phase noise and launched in the lattice. The first row depicts the band structure (bold black lines) and the position of the propagation constant of the soliton (dashed blue line). Solitons with a negative propagation constant are stable for many time steps. Solitons with a positive propagation constant, on the other hand, converge without any external perturbation into stable solitons (first row). All solitons show a robustness against external noise (row 1 to 4). Propagations were averaged over 1000 realizations. Noise amplitudes are given in multiples of 2π. In all cases, the nonlinear factor is fixed to $\Gamma = \pi$ which leads to an adoption of the soliton intensity according to its width.

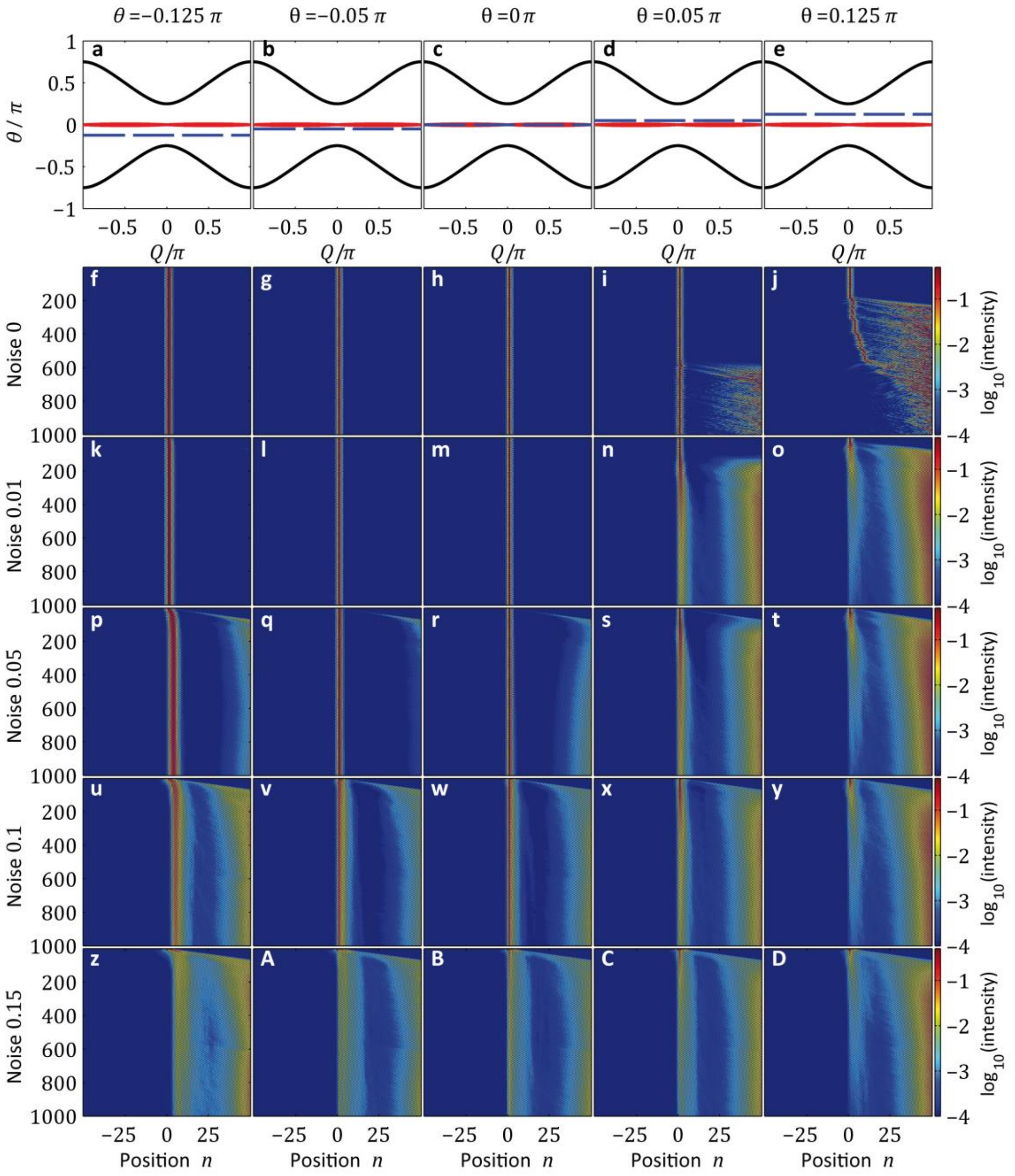

Supplementary Figure 8 | Qualitative stability analysis of the local $\mathcal{PT}$-symmetric solitons. Similar to Supplementary Fig. 7, numerically determined soliton profiles are perturbed by a phase noise and afterwards launched in the local $\mathcal{PT}$-symmetric lattice. The first row depicts the band structure (real part corresponds to the solid black line and the imaginary part to the solid red line) and the position of the propagation constant of the soliton (dashed blue line). Again, solitons with a positive propagation constant converge into stable solutions even without any external perturbation (first row). Note that this time the radiative emissions during this convergence are amplified along the gain direction. Absorbing boundaries are placed at position $n = \pm 192$. Although any perturbation is transported away from the soliton, higher noise levels lead to the decay of the soliton. In this case, again, noise amplitudes are given in multiples of 2π. Here, the nonlinear factor is $\Gamma = \pi$.

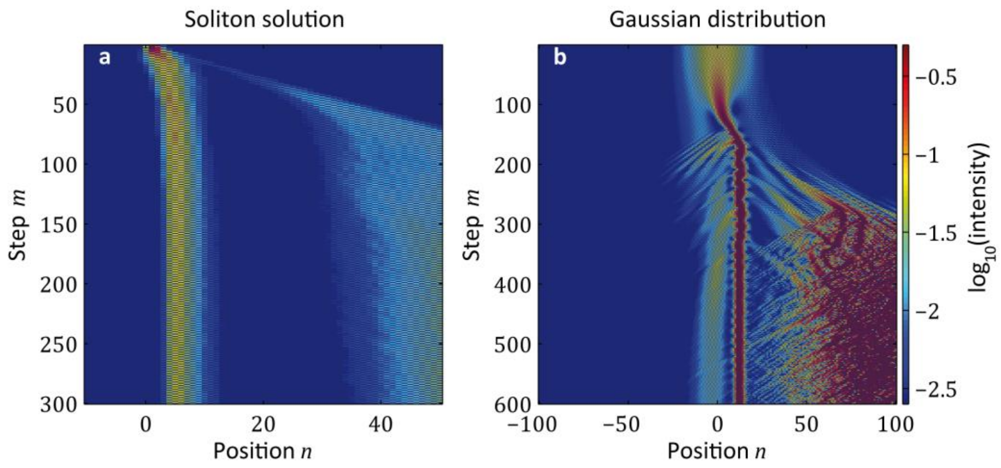

Supplementary Figure 9 | Simulation of the formation of a dissipative soliton. a, If a wide soliton with propagation constant $\theta = -0.125\pi$ is heavily perturbed with a phase noise with a distribution width of $0.1 \times 2\pi$ the soliton will emit the internal excitation and start to move into the gain direction. During this movement, the nonlinear wave is amplified and forms again a stationary soliton with an adapted width ($G = 1.1$). Same data is shown as in Supplementary Fig. 8. **b**, By starting with a broad distribution ($n_0 = 20$) the mismatch between soliton and initial lattice excitation is very large. The soliton is draught like the noisy wave in Supplementary Fig. 8 along the gain direction until it is sufficiently amplified to form a narrow soliton solution, which has lost its mobility. Here, a nonlinear factor of $\Gamma \approx 0.0025\pi$ is assumed.

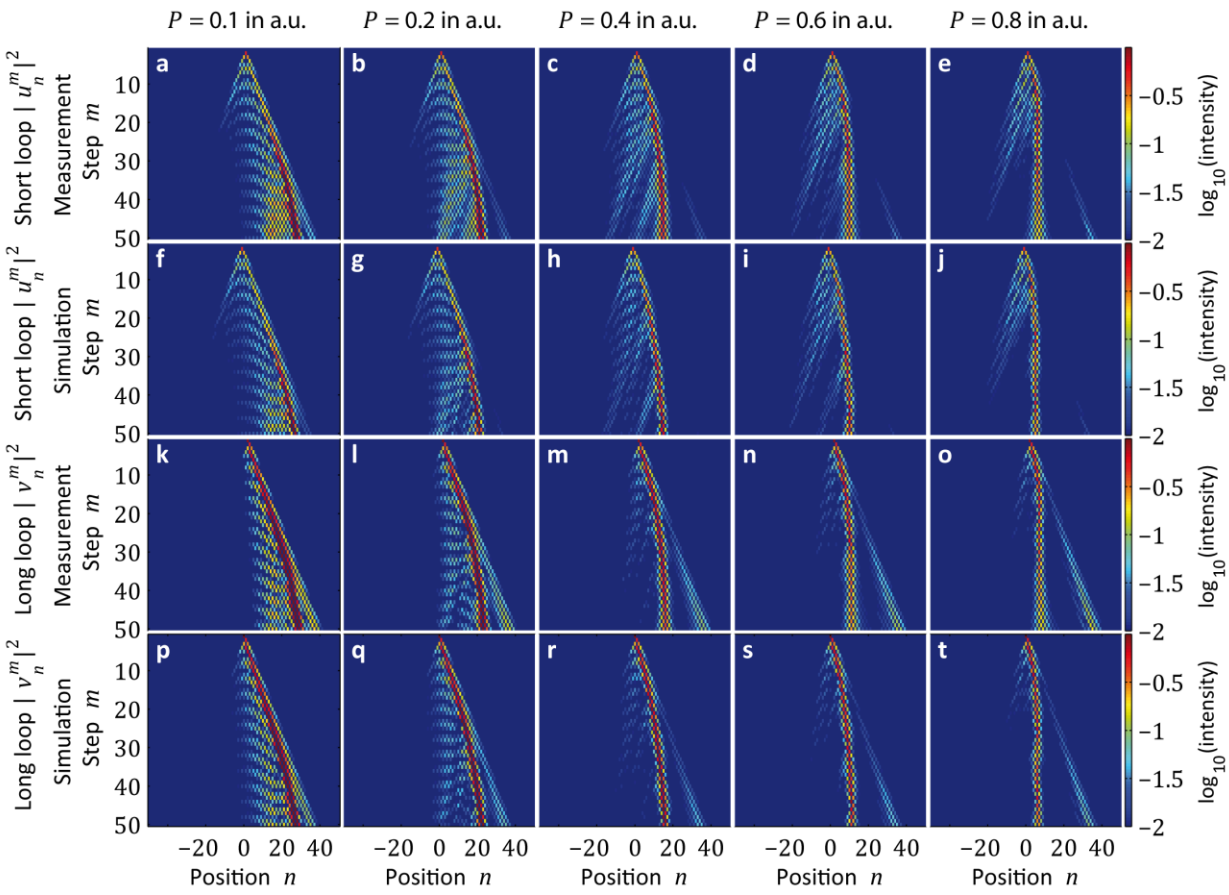

Supplementary Figure 10 | Simulations and measurements of the light walk in a locally $\mathcal{PT}$-symmetric lattice. Like in Supplementary Figs. 4, a single pulse initiated the experiment leading to a light walk like spreading. However, the pulses propagating on the local $\mathcal{PT}$–symmetric lattice to the left are attenuated, while the pulses propagating to the right are amplified ($G = 1.1$). The continuous increase in power leads to an accumulation of nonlinear phases and therefore to the bending of the sidearm already at linear and medium input power levels (first three columns). At higher input levels, the increase in power is sufficient for the nonlinear sidearm to stop completely resulting in the formation of the double discrete soliton as shown in Supplementary Figs.4. Due to the alternation of the soliton between long (gain) and short loop (loss) the soliton avoids any further increase in power and forms a stable state (fifth column). In the simulation the same nonlinear factor of $\Gamma \approx 0.84\pi$ like in Supplementary Figs. 4 was chosen for a comparison between both cases. Additionally the input powers in both cases (Supplementary Figs. 4 and Supplementary Figs. 10) have the same scaling.

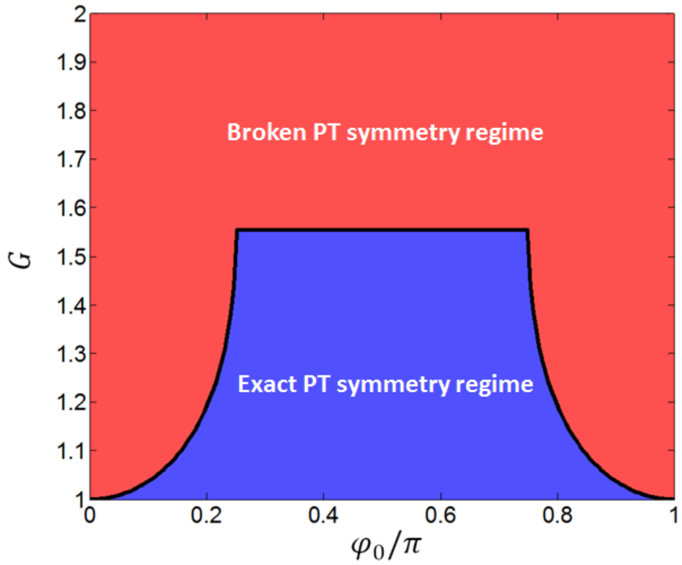

Supplementary Figure 11 | PT symmetry breaking threshold for different values of $\boldsymbol{\varphi_0}$ and $\boldsymbol{G}$. This map is obtained by solving equation (9) for the eigenvalue θ as a function of the transverse Bloch momentum Q. While in the blue area, θ is real for all values of Q, in the red area, θ is partially or entirely complex.

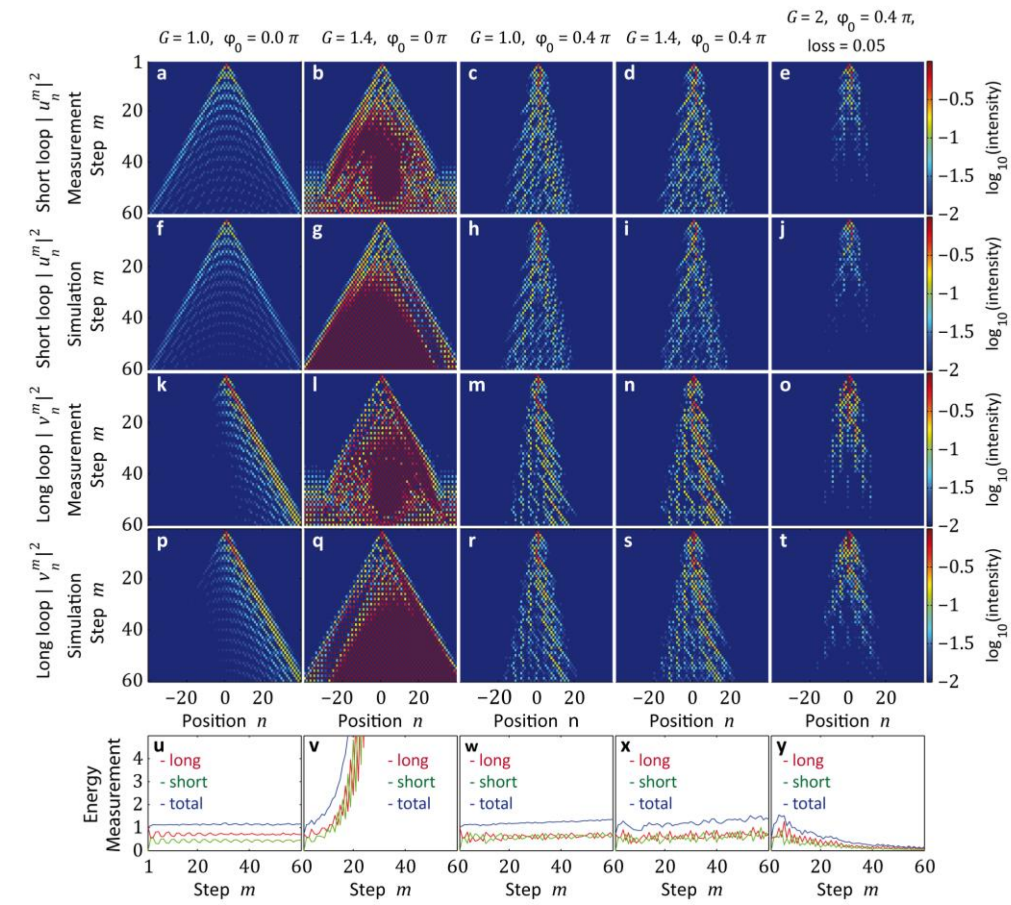

Supplementary Figure 12 | Simulations and measurements of the light walk spreading in a globally $\mathcal{PT}$-symmetric lattice in the linear regime. Again, like in Supplementary Figs. 4 and 10, the input state is formed by a single pulse. Without any modulation the light walk distribution is formed (first column), which preserves the energy (**u**). If an alternating gain/loss modulation is switched on, the system is above the $\mathcal{PT}$ threshold leading to an exponential increase of the fields during the propagation (second column) and also of the total energy (**v**). In contrast to this, a pure phase modulation conserves the energy (**w**) but reduces the mobility of the light on the lattice (third column). If both modulation schemes are combined, the system is below the $\mathcal{PT}$ threshold showing power oscillations but no exponential increase of the energy (**x**) or the fields (fourth column). The last column shows the saturable absorber in the linear limit as depicted in Fig. 7. Due to the additional loss of 5% per roundtrip the energy (**y**) and the fields (fifth column) are falling of rapidly. Because the detector in the short loop is placed before the acousto-optic modulator, which attenuates or amplifies the fields, each row of the simulated field of the short loop v_n^m has to be multiplied by $G^{\pm 1/2}$. The simulations were performed with a vanishing nonlinear factor $\Gamma = 0$.

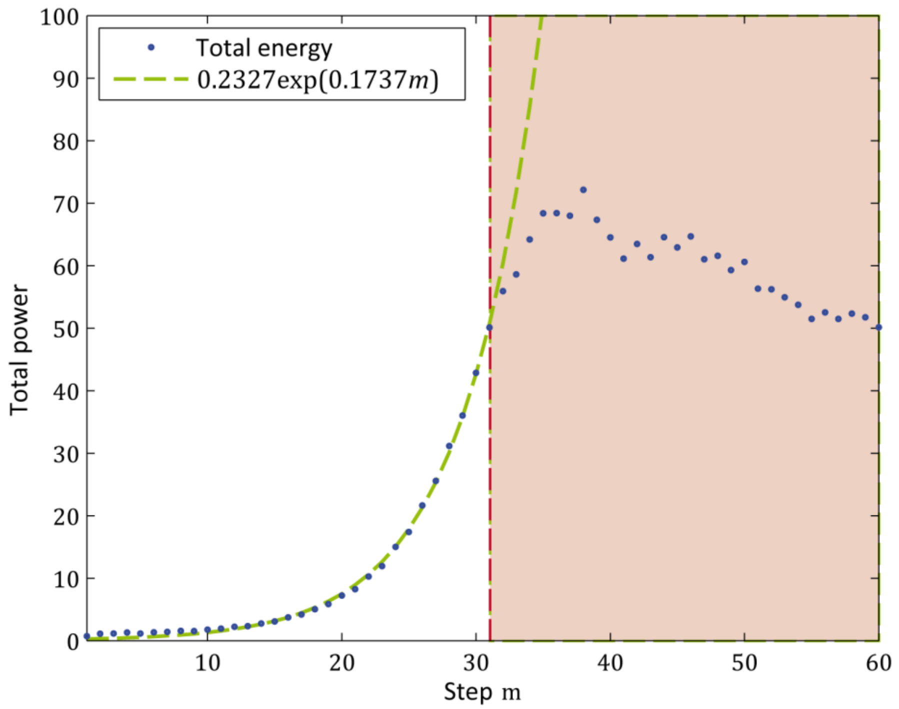

Supplementary Figure 13 | Comparison between the experimental data and theoretical expectations for the total power above the $\mathcal{PT}$ threshold. The same experimental data as in Supplementary Fig. 12(**v**) is shown with an adapted vertical axis for depicting the dependency of the total power on the time step m. Above the $\mathcal{PT}$ threshold, the eigenvalues of the system are complex, which leads in general to an exponential growth in power. Between step 1 and 31 an exponential growth of the form $P(m) = A \cdot \exp(Bm)$ perfectly fits to the measured data, with the estimated parameters $A \approx 0.2327$ and $B \approx 0.1737$. Due to the rapid growth above the threshold, the total power is already 60-fold at $m = 31$. At this point, the erbium doped fiber amplifiers saturate and can no longer support the exponential growth behavior. The power flattens and even decreases slightly due to the recovering of the amplifiers. The saturation of the amplifiers explains also the discrepancy between the measured and simulated propagation depicted in Fig. 4(**c**,**e**), Fig. 5(**a**,**c**), the second column of the Supplementary Fig. 12 and the second column of the Supplementary Fig. 14.

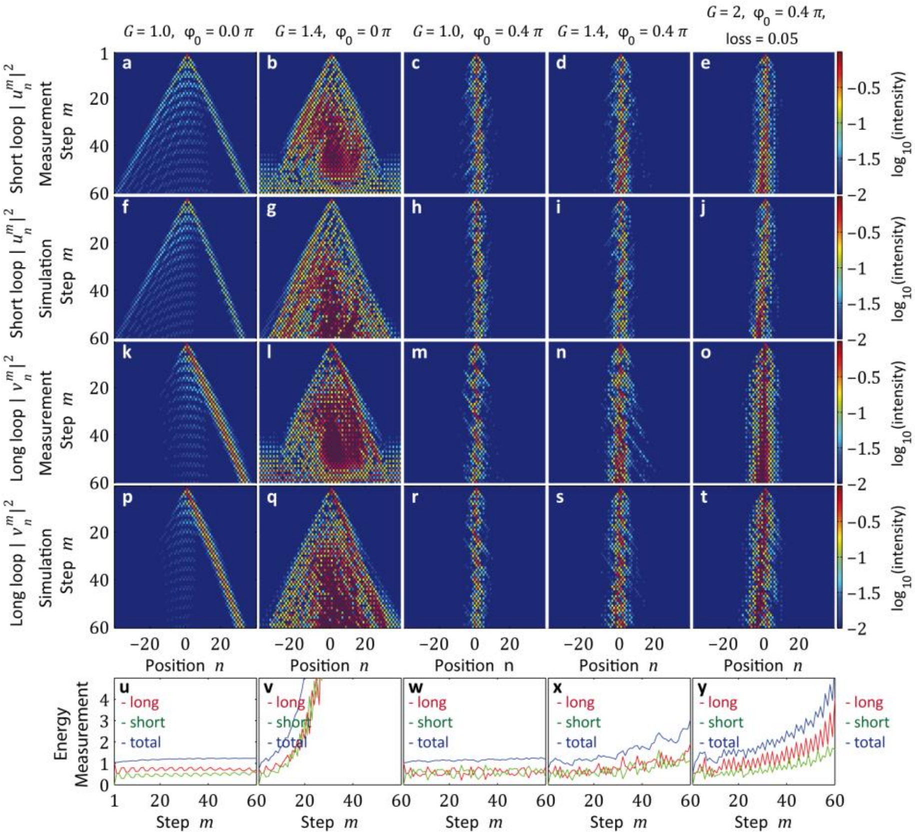

Supplementary Figure 14| Simulations and measurements of the light walk spreading in a globally $\mathcal{PT}$-symmetric lattice in the nonlinear regime. Same conditions as in the previous Supplementary Fig. 12, but with a three times higher input power level. The conservative system shows only a slight bending of the sidearm (first column) due to the rather low input power level. In addition, the system with the broken $\mathcal{PT}$ symmetry (second column) hardly shows a change compared to the linear regime (second column of the Supplementary Fig. 12). However in case of an active phase modulation the accumulation of nonlinear phases is already enough to prevent any spreading of the distribution (last three columns). The mismatch between the excitation of only one lattice point and the phase potential with a spatial periodicity of 4 positions leads to strong internal oscillations. **d**,**i**,**n**,**s**, depict clearly the localization on the $\mathcal{PT}$ lattice with a present amplitude and phase modulation. Due to the strict distinction between nonlinear phase accumulation and the modulation of the amplitude and phase, a very small continuous error is accumulated leading to a global gain. This error is comparable to the numeric errors arising in the split step method when solving the Nonlinear Schrödinger Equation. However, this idiosyncrasy can be utilized to compensate for a global loss of 5% and to form an effective saturable absorber (fifth column). Again each row of the simulated field of the short loop has to be multiplied by $G^{\pm 1/2}$. In the simulations, a nonlinear factor of $\Gamma \approx 0.26\pi$ was assumed.

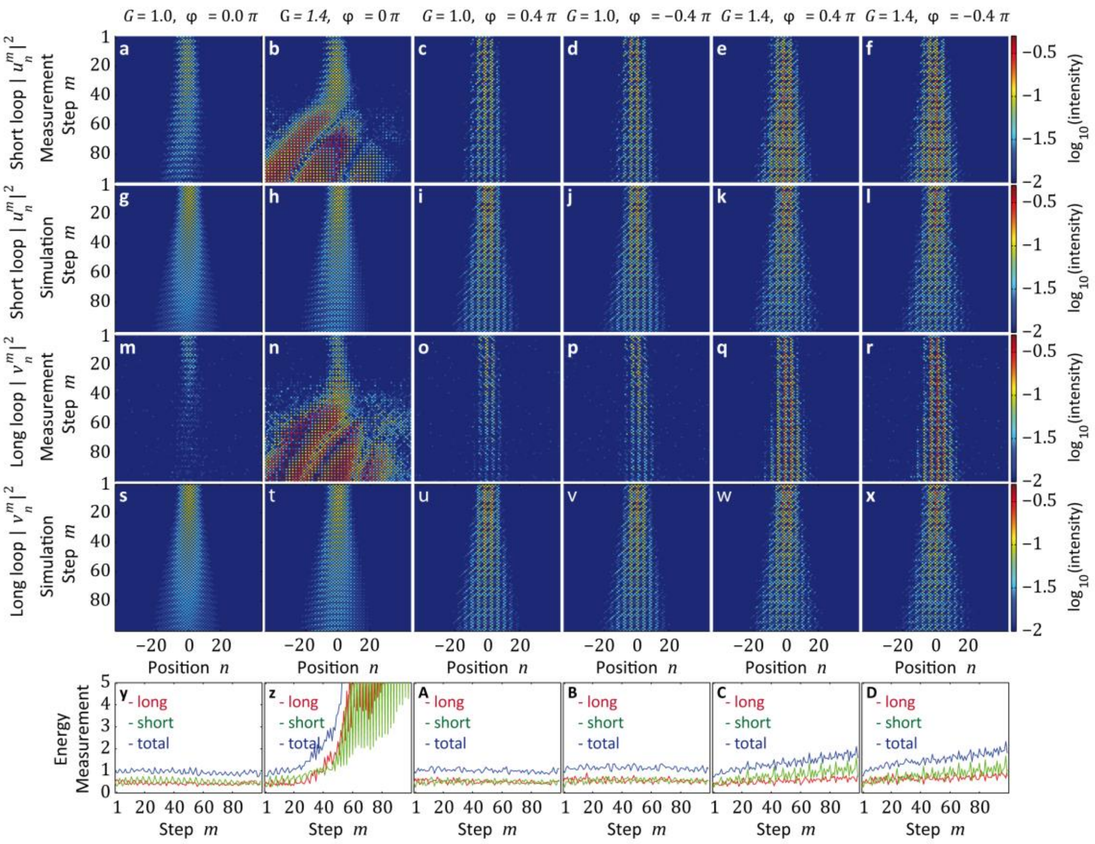

Supplementary Figure 15| Simulations and measurements of Gaussian distributions spreading in a globally $\mathcal{PT}$-symmetric lattice in the linear regime. In this measurement, a Gaussian distribution according to Supplementary Eq. 10 was generated with a width of $w = 7.3$ positions. Without any modulation, this distribution spreads similar to a Gaussian beam through a continuous medium (first column). Even if amplification and attenuation is switched on this behavior hardly alters due to the small spectral width of the excitation and the appearance of imaginary eigenvalues of θ at the edge of the Brillouin zone (second column) (see Fig. 5(a,c)). In the experiment however an accumulation of noise is visible (**b**,**n**,**z**). If the phase modulation is switched on, the propagation is quite similar to the evolution inside a waveguide array (third to sixth column). Depending on the sign of the phase modulation, the distribution starts either with a central dip (third and fifth column) or a hump (fourth and sixth column). The modulation of the pulse amplitudes has nearly no influence on the spreading on the lattice. The simulations where performed with a vanishing nonlinear factor.

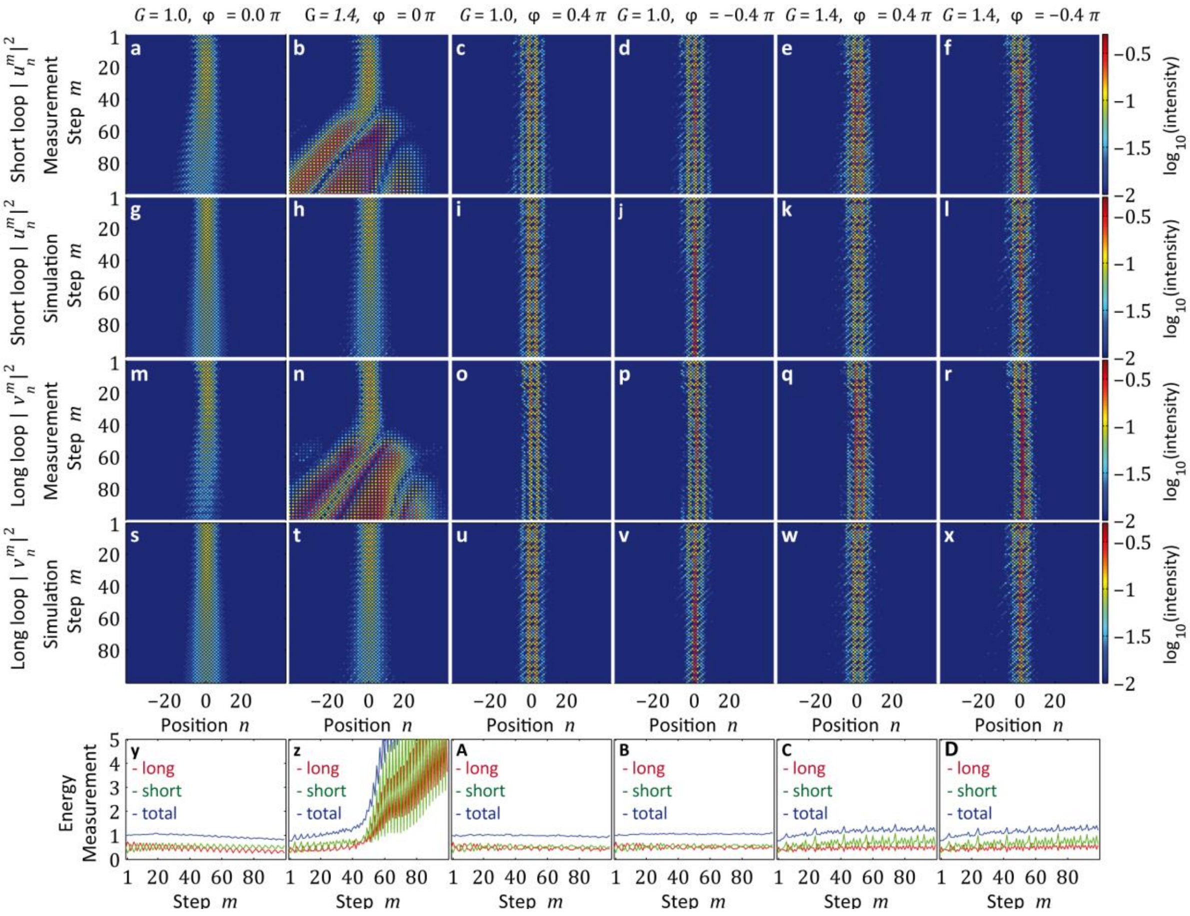

Supplementary Figure 16| Simulations and measurements of discrete $\mathcal{PT}$ solitons in a globally $\mathcal{PT}$-symmetric lattice. The same initial situation as in Supplementary Figs. 15 was used. The first column shows the formation of Schrödinger solitons (first column). In the measurement there was a slight depletion of the energy, which leads to the spreading of the profile (**a**,**m**). Similar to Supplementary Figs. 15 (second column) the broken $\mathcal{PT}$ phase has hardly an influence on the propagation of the solitons (second column) except for the increase in noise in the experimental system (**b**,**n**). With an applied phase potential discrete solitons form in all of the remaining cases. The inversion of the sign of the phase modulation switches between single hump (third and fifth column) and double hump solitons (fourth and sixth column). Even in presence of gain and loss below the $\mathcal{PT}$ threshold, a stable propagation of the solitons is visible for about 100 roundtrips (fifth and sixth column). In the simulations, a nonlinear factor of $\Gamma \approx 0.011\pi$ and a beam width of $n_0 \approx 7.3$ positions was assumed.

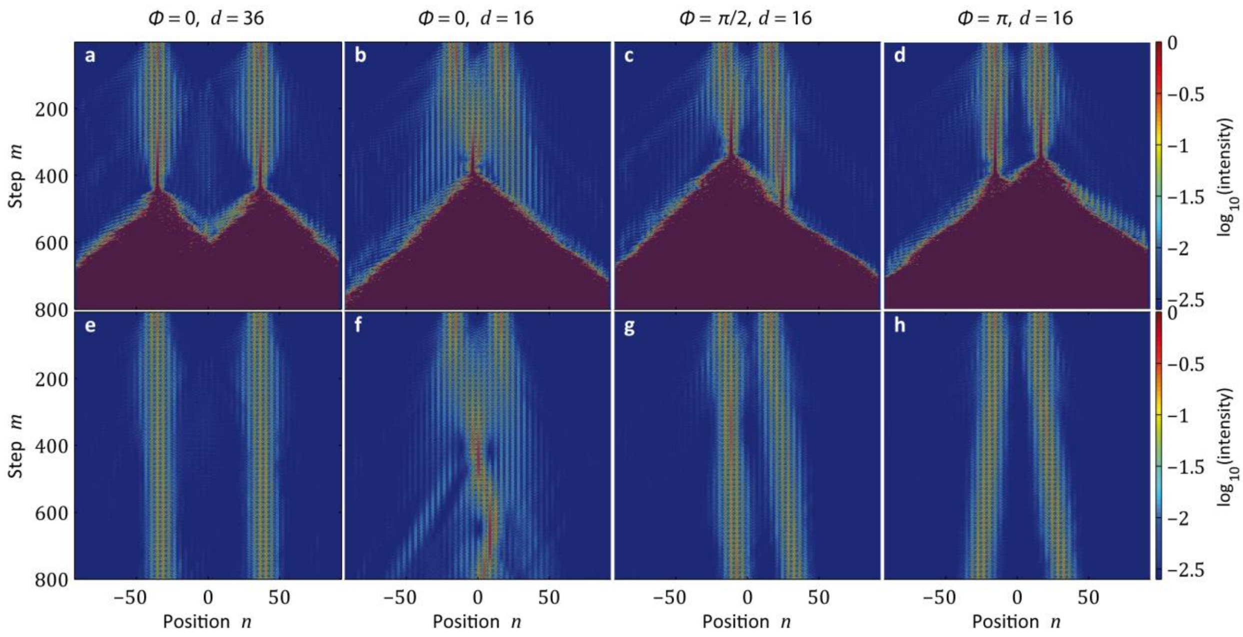

Supplementary Figure 17| Simulation of the interaction of discrete $\mathcal{PT}$ solitons in a globally $\mathcal{PT}$-symmetric lattice. The first row shows the interaction of $\mathcal{PT}$ solitons, while the second row depicts conservative solitons for comparison. The $\mathcal{PT}$ solitons were simulated with a nonlinear coefficient of $\Gamma \approx 0.002\pi$ and the conservative solitons with a slightly higher coefficient of $\Gamma \approx 0.0025\pi$. A relative phase of $\Phi = 0$ between both solitons leads to an attractive force (**a,b,e,f**). Depending on the initial distance d of the two solitons, the interaction is either vanishing $d = 36$ (**a,e**) or visible $d = 16$ (**b-d,f-h**). For a relative phase of $\Phi = \pi/2$ an energy transfer between both solitons takes place resulting in a slight oscillation in the intensity profile. In case of a phase shift of $\Phi = \pi$, a repulsive interaction between both solitons is established. In these simulations, the following parameters were used for the gain/loss and phase modulations respectively: $G = 1.4$ and $\varphi_0 = 0.4\pi$.

Supplementary Notes

Supplementary Note 1: Discussion of the evolution equations

In former experiments (see Supplementary Ref. 4) the following set of equations

$$\begin{aligned} \text{short loop:}\ u_n^{m+1} &= \tfrac{1}{\sqrt{2}}\left(u_{n+1}^m \exp(i\Gamma\, |u_{n+1}^m|^2) + i v_{n+1}^m \exp(i\Gamma\, |v_{n+1}^m|^2)\ \right) \exp\big(i\varphi(n)\big) \\ \text{long loop:}\ \ v_n^{m+1} &= \tfrac{1}{\sqrt{2}}\left(i u_{n-1}^m \exp(i\Gamma\, |u_{n-1}^m|^2) + v_{n-1}^m \exp(i\Gamma\, |v_{n-1}^m|^2)\ \right), \end{aligned} \tag{1}$$

was used to describe the evolution of a conservative system without any gain and loss. In this model (see Supplementary Fig. 2(a)) the pulses first acquire a nonlinear phase shift proportional to $u_n^m \exp(i\Gamma|u_n^m|^2)$ and $v_n^m \exp(i\Gamma|v_n^m|^2)$. Afterwards, both loops are coupled with a ratio of 50/50 and finally a linear phase shift $\exp\big(i\varphi(n)\big)$ is applied to the short loop (turquois phase modulator). However, there is no difference between applying first a nonlinear phase shift and afterwards a linear phase shift and vice versa. Therefore, the phase modulator may be shifted towards the end of the short loop (magenta phase modulator in Supplementary Fig. 2(a)), which is the actual position in the experiment. Depending on whether the coupler is inserted before or after the DCF, the evolution equations can also take the form of

$$\begin{aligned} \text{short loop:}\ u_n^{m+1} &= \frac{1}{\sqrt{2}}(u_{n+1}^m + i v_{n+1}^m)\exp(i\Gamma(|u_{n+1}^m + i v_{n+1}^m|^2)/2\ ) \exp\big(i\varphi(n)\big) \\ \text{long loop:}\ \ v_n^{m+1} &= \frac{1}{\sqrt{2}}(i u_{n-1}^m + v_{n-1}^m)\exp(i\Gamma(|i u_{n-1}^m + v_{n-1}^m|^2)/2\ ), \end{aligned} \tag{2}$$

for the latter case, while the described dynamic and the physical meaning is the same.

In case of the $\mathcal{PT}$ symmetric system (see Supplementary Fig. 2(b)), one has to pay great attention to the ordering since amplification/attenuation and the nonlinear phase shift do not commute any more. In experiment, a single pulse is launched into the long loop directly before the 50/50 coupler. After the splitting the pulses acquire a nonlinear phase shift in each loop. At the end of one roundtrip, the pulses are amplified/attenuated and manipulated by the phase modulator leading to the final evolution equations

$$\begin{aligned} u_n^{m+1} &= \frac{\sqrt{G_\mathrm{u}}}{\sqrt{2}}(u_{n+1}^m + i v_{n+1}^m) \cdot \mathrm{e}^{\frac{i\Gamma}{2}\left(|u_{n+1}^m + i v_{n+1}^m|^2\right)} e^{i\varphi_n}, \\ v_n^{m+1} &= \frac{\sqrt{G_\mathrm{v}}}{\sqrt{2}}(v_{n-1}^m + i u_{n-1}^m) \cdot \mathrm{e}^{\frac{i\Gamma}{2}\left(|v_{n-1}^m + i u_{n-1}^m|^2\right)}, \end{aligned} \tag{3}$$

used in the main part (see Eq. 1).

In conclusion, both pictures describe accurately the dynamics on the mesh lattices. However while the first evolution equation (Eq. 1) holds only for Hermitian systems without gain and loss, the more general Eq. 3 has to be used for the description of dissipative systems where the interplay between amplification/attenuation and nonlinearity is important and cannot be exchanged.

Supplementary Note 2: Band structures of the mesh lattices

The band structures of the double discrete mesh lattices (see Supplementary Figs. 3(a-c)) can be obtained by inserting a plane wave ansatz

$$\begin{pmatrix} u_n^m \\ v_n^m \end{pmatrix} = \begin{pmatrix} U \\ V \end{pmatrix} e^{i\theta m} e^{iQn} \qquad (4)$$

in the evolution equations Eqs. 1 of the linear systems. The parameter Q can be seen as a transversal wavenumber or a Bloch momentum, while θ stands for the longitudinal wavenumber, which is the propagation constant of the plane wave. For the passive mesh lattice ($G = 1, \varphi = 0$) this ansatz yields the dispersion relation

$$\cos\theta = \frac{1}{\sqrt{2}} \cos Q \qquad (5)$$

between the longitudinal and transversal wavenumber θ and Q (see Supplementary Fig. 3(d)). By amplifying the pulses traveling to the right and attenuating the pulses into the opposite direction a local $\mathcal{PT}$-symmetric lattice is generated, which has a complex dispersion relation (see Supplementary Fig. 3(b,e))

$$\cos\theta = \frac{1}{\sqrt{2}} \cos\left(Q - \frac{i}{2} \ln G\right). \qquad (6)$$

For deriving this band structure, the same method based on Supplementary Eq. 4 as before in the conservative case can be used.

The imaginary part arising for $G \neq 1$ leads to an exponential growth for the initial wave. By assuming a finite size system with absorbing boundaries, one can prevent the growth of an initial distribution. This distribution is amplified as long as it moves into the direction of the gain (the distribution has to stay inside the fiber loop, which has an effective amplification) but when it reaches the boundary of the system, it gets reflected and moves from this point on into the loss direction. Due to this inevitable shift in direction, a wave cannot grow indefinitely in a finite size system in contrast to the boundless growth in the infinite system. Formally, one can regain a dispersion relation supporting real eigenvalues by additionally applying a transformation $\sim G^{-n/2}$ to the ansatz in Supplementary Eq. 4.

While this ansatz holds for the first two lattices adaptions have to be made for the global $\mathcal{PT}$-symmetric case (see Supplementary Fig. 3(c)). Owing to the 4 positions wide phase potential (see Supplementary Fig. 6)

$$\varphi_n = \begin{cases} +\varphi_0, & \mathrm{mod}(n+3,4) < 2 \\ -\varphi_0, & \text{otherwise.} \end{cases} \qquad (7)$$

in the $\mathcal{PT}$-symmetric case the ansatz in Supplementary Eq. 4 doesn't cover any more the complete unit cell. Therefore, a modification is needed taking all sublattices of the unit cell into account. With respect

to the temporal and spatial periodicity of the lattice, the following set of 14 equations describes the full dynamic inside the unit cell:

$$U_n^{m+1} e^{i\theta} = \frac{e^{+\frac{1}{2}\ln(G)}}{\sqrt{2}} e^{iQ} (U_{n+1}^m + i V_{n+1}^m), \quad (8.1)$$
$$V_n^{m+1} e^{i\theta} = \frac{e^{-\frac{1}{2}\ln(G)}}{\sqrt{2}} e^{-iQ} (V_{n+3}^m + i U_{n+3}^m) e^{-i\varphi_0}, \quad (8.2)$$

$$U_{n+1}^{m+1} e^{i\theta} = \frac{e^{+\frac{1}{2}\ln(G)}}{\sqrt{2}} e^{iQ} (U_{n+2}^m + i V_{n+2}^m), \quad (8.3)$$
$$V_{n+1}^{m+1} e^{i\theta} = \frac{e^{-\frac{1}{2}\ln(G)}}{\sqrt{2}} e^{-iQ} (V_n^m + i U_n^m) e^{+i\varphi_0}, \quad (8.4)$$

$$U_{n+2}^{m+1} e^{i\theta} = \frac{e^{+\frac{1}{2}\ln(G)}}{\sqrt{2}} e^{iQ} (U_{n+3}^m + i V_{n+3}^m), \quad (8.5)$$
$$V_{n+2}^{m+1} e^{i\theta} = \frac{e^{-\frac{1}{2}\ln(G)}}{\sqrt{2}} e^{-iQ} (V_{n+1}^m + i U_{n+1}^m) e^{+i\varphi_0}, \quad (8.6)$$

$$U_{n+3}^{m+1} e^{i\theta} = \frac{e^{+\frac{1}{2}\ln(G)}}{\sqrt{2}} e^{iQ} (U_n^m + i V_n^m), \quad (8.7)$$
$$V_{n+3}^{m+1} e^{i\theta} = \frac{e^{-\frac{1}{2}\ln(G)}}{\sqrt{2}} e^{-iQ} (V_{n+2}^m + i U_{n+2}^m) e^{-i\varphi_0}, \quad (8.8)$$

$$U_n^m e^{i\theta} = \frac{e^{-\frac{1}{2}\ln(G)}}{\sqrt{2}} e^{iQ} (U_{n+1}^{m+1} + i V_{n+1}^{m+1}), \quad (8.9)$$
$$V_n^m e^{i\theta} = \frac{e^{+\frac{1}{2}\ln(G)}}{\sqrt{2}} e^{-iQ} (V_{n+3}^{m+1} + i U_{n+3}^{m+1}) e^{-i\varphi_0}, \quad (8.10)$$

$$U_{n+1}^m e^{i\theta} = \frac{e^{-\frac{1}{2}\ln(G)}}{\sqrt{2}} e^{iQ} (U_{n+2}^{m+1} + i V_{n+2}^{m+1}), \quad (8.11)$$
$$V_{n+1}^m e^{i\theta} = \frac{e^{+\frac{1}{2}\ln(G)}}{\sqrt{2}} e^{-iQ} (V_n^{m+1} + i U_n^{m+1}) e^{+i\varphi_0}, \quad (8.12)$$

$$U_{n+2}^m e^{i\theta} = \frac{e^{-\frac{1}{2}\ln(G)}}{\sqrt{2}} e^{iQ} (U_{n+3}^{m+1} + i V_{n+3}^{m+1}), \quad (8.13)$$
$$V_{n+2}^m e^{i\theta} = \frac{e^{+\frac{1}{2}\ln(G)}}{\sqrt{2}} e^{-iQ} (V_{n+1}^{m+1} + i U_{n+1}^{m+1}) e^{+i\varphi_0}, \quad (8.14)$$

Again these equations can be solved by a Floquet Bloch ansatz in the form of Supplementary Eg. 4 leading to the dispersion relation (see Supplementary Fig. S3(f))

$$\cos(4Q) = 8\cos^2(2\theta) + 8\cosh(\ln(G))\cos(\varphi_0)\cos(2\theta) + \cosh(2\ln(G)) + 4\cos^2(\varphi_0) - 4 \quad (9)$$

of the $\mathcal{PT}$ mesh lattice. In contrast to the size of the Brillouin zone of the passive and local $\mathcal{PT}$-symmetric lattice ranging from $-\pi$ to π for θ and Q, the Brillouin zone of the $\mathcal{PT}$ system is limited to $Q = -\pi/4\,..\,\pi/4$ and $\theta = -\pi/2\,..\,\pi/2$.

Supplementary Note 3: Reduction of the mesh lattice

Since in each step, pulses can only move from a position n to the neighboring positions $n+1$ and $n-1$, every second position in the lattice is not occupied (see Supplementary Fig. 5(a)). When numerically searching for soliton solutions, one can assume a stationary profile over a double step. Another possibility is to compress the mesh lattice. In this case, every second row is moved upwards to the unoccupied positions of the sublattice (see Supplementary Fig. 5(b)). By demanding a stationary profile for the complete row over one time step, the whole evolution of the double step system is taken into account. For the numerical determination of the soliton profiles, it turns out that the compressed lattice features a higher robustness and allows a faster calculation.

Supplementary Note 4: Stability of solitons and convective instability on the local $\mathcal{PT}$-symmetric lattice

In what follows, we provide a qualitative stability analysis of the solitons observed in the local $\mathcal{PT}$-symmetric lattice. The soliton profiles are calculated by solving the nonlinear evolution equations with Newton's algorithm for different propagation constants (see Supplementary Fig. 7 first row). Such solitary solutions are then launched to propagate for 1000 time steps. In case of no amplification and attenuation, all solitons with a propagation constant $\theta \leq 0$ are stable (see Supplementary Fig. 7 second row). Solitons with a positive propagation constant, on the other hand, converge into a stable solution, even if they are not externally perturbed. We then perturb all soliton solutions by an equally distributed phase noise. The noise amplitude is varied from 0 up to $0.15 \times 2\pi$ featuring heavy perturbations. For small noise amplitudes, internal oscillations are excited.

By switching on the gain and loss, we next consider the local $\mathcal{PT}$-symmetric system. In this scenario, all pulses traveling to the right are amplified, and at the same time, all pulses that propagate towards the opposite direction are attenuated. While a complete one-parameter family of soliton solutions can be calculated, again solitons with a positive propagation constant tend to be unstable and converge into a stationary distribution even in the absence of any external perturbation (see Supplementary Fig. 8(i)).

In presence of an external perturbation, the added noise is radiated away from the center. Due to the dissipative lattice, this emission is amplified while propagating to the right. In an infinite system, this emission would continuously grow in power. However, in a finite size system, the amplified emission will reach the right boundary and be reflected into the loss direction. A propagation towards the initial soliton distribution is hindered due to the attenuation. The total power of the system cannot further increase because of the reflection at the boundaries. In this way, the system is stabilized since the total power is limited.

By starting with a wide soliton, perturbations can lead to a tremendous emission of radiation. At the same time, the decrease of the soliton power leads to a moving solution. While this distribution is draught to the gain direction, it is again amplified until a new soliton solution is formed, which seems to be in general smaller compared to the initial soliton (see Supplementary Fig. 9(a)). This intriguing effect is clearly visible, when a broad Gaussian distribution is launched. In this case, the mismatch between the initial distribution and the final soliton profile leads to a long-lasting acceleration until the distribution has acquired enough power to form a soliton (see Supplementary Fig. 9(b)). By starting with a Gaussian distribution only the smallest soliton can be excited.

Supplementary Note 5: Interaction of global $\mathcal{PT}$ solitons

For a further characterization of $\mathcal{PT}$ solitons, the interaction of two nonlinear waves is investigated. Due to the strict separation of gain and loss and nonlinear propagation, $\mathcal{PT}$ solitons on double discrete mesh lattices tend to show an intrinsic instability. While this phenomenon is utilized for the saturable absorber, it leads in general to the explosion of $\mathcal{PT}$ solitons in presence of amplification and attenuation (see Supplementary Fig. 17(a)) while the conservative counterparts (vanishing imaginary potential) are stable (see Supplementary Fig. 17(e)). If both solitons are launched next to each other (see Supplementary Fig. 17(b-d,f-h)) the interaction of the solitons depend on the relative phase Φ between the two distributions. A vanishing relative phase leads to an attractive interation between both solitons (see Supplementary Fig. 17(b,f)), which leads to a merging of the two solitons. As in case of the separated solitons, the dynamic is again dominated by the intrinsic instability, which is triggered faster due to the increased power levels of the merged soliton. By assuming a relative phase of $\Phi = \pi/2$, an energy transfer between both solitons takes place, leading to a slight oscillation of the intensities (see Supplementary Fig. 17(c,g)).The initial transfer of energy from the right soliton to the left soliton leads to an explosion of the latter one and hinders the instability of the right soliton. In the last case (see Supplementary Fig. 17(d,h)), a repulsive interaction is simulated by choosing an overall phase of $\Phi = \pi$. Again, after merging of the two solitons, instabilities set an end to the soliton propagation.

Supplementary Methods

Experimental setup

Linear and nonlinear versions of the fiber-loop setup depicted in Supplementary Fig. 1 have been reported in Refs. [1-4]. These four references (which are all accompanied by supplementary material) contain detailed explanations on experimental methods as well as the time-multiplexing approach.

The basic approach is to use two fiber loops of different length to implement a so called time multiplexing scheme [5]. The average length of the fiber is $L = 4.3\ \mathrm{km}$, while the length difference is only $\Delta L = 45\ \mathrm{m}$. Both loops are connected by a 50/50 coupler. Additional couplers are utilized for injecting the initial pulse and for monitoring the pulse distributions in both loops.

In this arrangement, only those pulses that have traveled for an equal number of times along the short and the long loop can simultaneously arrive at the central 50/50 coupler. Because of this interesting property, any low frequency noise, that may take place on time scales much longer than the measurement time, has no influence on the results. Therefore, the experimental setup acquires an intrinsic stability against acoustic and thermal environmental effects.

In order to maintain this stability, all measurements have to start with the same seed pulse. For this purpose, by employing a Mach-Zehnder modulator, a chain of 50 ns pulses is produced from a CW signal emitted from a distributed feedback laser diode operating at $\lambda_{\mathrm{signal}} = 1555\ \mathrm{nm}$. The chain of pulses is then amplified with an erbium-doped fiber amplifier (EDFA). An acousto-optic modulator (AOM) is used to suppress the background between two adjacent pulses and another AOM is utilized to select individual pulses from this sequence. Before injecting the seed pulse into the fiber loop system, through a tunable bandpass filter, the amplified spontaneous emission noise of the EDFA is removed from the pulse. Finally, the pulse is inserted in the long loop via an additional 50/50 coupler which is also used for monitoring the pulse distribution.

Both loops are built up in a mostly symmetric way. At the beginning of each loop, in order to stabilize the amplification factor of the successive EDFA, a CW pilot signal at a wavelength of $\lambda_{\mathrm{pilot}} = 1535\ \mathrm{nm}$ is inserted. The amplifiers in the loop system are utilized to compensate for all losses that occur in one roundtrip except for the 3 dB loss at the central 50/50 coupler. In addition, two tunable bandpass filters are used to clean the pulses, which are then launched into the about 4 km long dispersion compensated fibers (DCF, type: OFS-HSDK, [6]) with a nonlinear parameter of $\gamma = 7\ (\mathrm{W{\cdot}km})^{-1}$. The effective length of these fibers is $L_{\mathrm{eff}} \approx 3\ \mathrm{km}$, which leads to a nonlinear phase shift of 2π at a peak power of about 300 mW. An additional single mode fiber spool is employed to generate the length difference of 45 m needed for the time multiplexing scheme.

For monitoring the pulse distribution in the short loop a 90/10 fiber coupler is used. A phase modulator in the short loop is controlled by an arbitrary waveform generator and establishes the phase potential defined by Eq. 6 of the main text. In addition to this phase modulation, an effective amplification or attenuation is needed to create the $\mathcal{PT}$-symmetric environment. This is achieved by utilizing two AOMs

operating in zeroth order, which are normally set to 50% transmission. By increasing (decreasing) the transmission an effective gain (loss) is achieved. To prevent any back reflections from the AOMs isolators are installed in both loops.

In experiment, it is very important that the EDFAs keep a constant amplification factor throughout the whole measurement. This is ensured by continuously inserting pulses into the fiber loops before the measurement starts. These pulses are prevented from circulating through the loops by enforcing zero transmission in the AOMs, which are used inside the loops. At the beginning of the measurement, the AOM at the entrance of the two loop system closes right after inserting the seed pulse. Next, the AOMs inside the fiber loops are opened again to unblock the pulse circulation. After each set of measurements, the loop AOMs are closed again and the entrance AOM clears the way for the next seed pulse.

For measurements, which require a Gaussian distribution, a seed pulse is first inserted into the fiber loop system. Afterwards, all pulses inside the short loop are cleared after every other roundtrip. This modulation scheme generates a Gaussian distribution, which is an eigenstate of the system at the edge of the Brillouin zone and populates both bands. By employing the phase modulator one can shift the excitation from the edge towards the center and select one specific band. A detailed description of this procedure is given in the Supplementary Material of Ref. [4].

Comparison between simulations and experiments

The measurements, which are presented in the main text, are compared to the simulations based on the evolution equations Eqs. 1. Measurements and simulations are starting with a single pulse. Both in the main part and in the Supplementary Material they are normalized so that the total intensity in the second row of each figure is equal to 1/2. In case of a broad distribution, the initial state (first row) has a total intensity of 1/2 in each loop. For a better visualization, the propagations are shown in a logarithmic color scale.
For an excitation with a broad Gaussian distribution (see Figs. 6, Supplementary Figs. 15-17) the upper band of the conservative system was chosen. Excitations of this band are focused under the influence of the nonlinear self-phase modulation. In the simulation the initial distribution was assumed as

$$u_n^1 \sim e^{-\frac{n^2}{w^2}} \text{ and } v_n^1 \sim -e^{-\frac{n^2}{w^2}}, \qquad (10)$$

where $w \approx 7.3$ stands for the width of the distribution and was chosen for a best fit to the experimental data.

Nonlinearity in experiment and simulations

In the experiment, two approximately 4 km long dispersion compensated fibers (DCF) are used, due to their small modal field diameter and hence high intensities inside the fiber core. As a result, the effective nonlinear factor of the fiber $\gamma \approx 7\ (\mathrm{W\cdot km})^{-1}$ is much higher, compared to standard single mode fibers $\gamma \approx 1\ (\mathrm{W\cdot km})^{-1}$ [6]. In order to estimate the nonlinear phase shift acquired during one roundtrip, the effective length of the fibers [6,7] is assumed to be

$$L_{\mathrm{eff}} = \frac{1 - e^{-0.23\alpha L}}{0.23\alpha} \approx 3\ \mathrm{km}, \quad (11)$$

with the attenuation $\alpha \approx 0.6\mathrm{dB/km}$ [6]. The power dependent nonlinear phase shift [6,7] can then be expressed as

$$\varphi_{\mathrm{nonlinear}} = \frac{2\pi n_2}{\lambda A_{\mathrm{eff}}} L_{\mathrm{eff}} P_{\mathrm{in}}, \quad (12)$$

where the nonlinear refractive index n_2 and the effective field area A_{eff} are taken from [6].

Therefore, a nonlinear phase shift of 2π is accumulated at a peak power of about 300 mW. While in the experiment the nonlinearity of the fiber is a fixed parameter, the peak power of the seed pulse is swept by an variable optical screw attenuator and an acousto-optical modulator to scale the total nonlinear phase shift. In this way, the amplitude of the distribution can be easily set as a percentage of the maximum available amplitude, which is about 150mW in the experiment.

In the experiment, the peak power in the fibers can only be estimated due to different limitations and imperfections. For example, directly measuring the peak power in the fiber would demand an additional monitor coupler at the beginning of the fiber. This, however, requires an additional splice between monitor coupler and DCF which results in an unknown loss. However, the power values can be deduced from simulations, due to the great agreement between experimental and numerical results. By adopting the nonlinear coefficient for a best fit of the simulation to the experiment, the maximum nonlinear phase shift can roughly be estimated, which on the other hand, allows the calculation of P_{in} based on Supplementary Eq. 12. Using this approach, the estimations of the peak power in the fibers were calculated, which label the power values in Figs. 2, 3 and 7 of the main paper.

In the simulations of the evolution equation, on the other hand, we kept the amplitudes fixed and instead the nonlinear coefficient Γ was changed. Note that in general the evolution is only influenced by the acquired self phase modulation, which is given by the product of the pulse intensity and the nonlinearity of the fiber (see Supplementary Eq. 12). Therefore, both approaches are identical and allow for a comparison between simulation and experiment, by adopting the nonlinear coefficient Γ in the simulation to a best fit to the experimental results. In the simulations presented in this work, the initial distributions are normalized and thus, besides experimental imperfections, no systematic difference occurs.

The solver described in the method section searches for solutions of the nonlinear evolution equations by varying the real and the imaginary parts of the initial distribution, thus inevitably changing the amplitude. Since the amplitude, width and the propagation constant of the soliton are correlated parameters, the nonlinear coefficient Γ was set to a fixed value of π in order to eliminate any further degrees of freedom. In this case again, due to the scaling of the nonlinear self-phase modulation, a comparison between the solver and the experiment or the simulations is possible.

Supplementary References

1. Regensburger, A. *et al.* Photon Propagation in a Discrete Fiber Network: an interplay of coherence and losses. *Phys. Rev. Lett.* **107,** 233902 (2011).

2. Regensburger, A. *et al.* Parity–time synthetic photonic lattices. *Nature* **488,** 167–171 (2012).

3. Regensburger, A. *et al.* Observation of defect states in PT-Symmetric optical lattices. *Phys. Rev. Lett.* **110,** 223902 (2013).

4. Wimmer, M. *et al.* Optical diametric drive acceleration through action–reaction symmetry breaking. *Nat. Phys.* **9,** 780-784 (2013).

5. Schreiber, A. *et al.* Photons Walking the line: a quantum walk with adjustable coin operations. *Phys. Rev. Lett.* **104,** 050502 (2010).

6. Gruner-Nielsen, L. *et al.* Dispersion-compensating fibers. *J. Lightwave Technol.* **23,** 3566–3579 (2005).

7. Agrawal, G. P. *Nonlinear Fiber Optics*. (Academic Press, Boston, 2007).
